# Antimicrobial activity of compounds identified by artificial intelligence discovery engine targeting enzymes involved in *Neisseria gonorrhoeae* peptidoglycan metabolism

**DOI:** 10.1186/s40659-024-00543-9

**Published:** 2024-09-05

**Authors:** Ravi Kant, Hannah Tilford, Camila S. Freitas, Dayana A. Santos Ferreira, James Ng, Gwennan Rucinski, Joshua Watkins, Ryan Pemberton, Tigran M. Abramyan, Stephanie C. Contreras, Alejandra Vera, Myron Christodoulides

**Affiliations:** 1 Neisseria Research Group, Molecular Microbiology, School of Clinical and Experimental Sciences, Faculty of Medicine, University of Southampton, Southampton, England SO16 6YD; 2 Medical Biotechnology Laboratory, Dr. B. R. Ambedkar Center for Biomedical Research, University of Delhi, North Campus, Delhi, 110007 India; 3 Programa de Pós-Graduação em Ciências da Saúde: Infectologia e Medicina Tropical, Faculdade de Medicina, Universidade Federal de Minas Gerais, Belo Horizonte, Minas Gerais 30130-100 Brazil; 4 Laboratory of Pathophysiology, Butantan Institute, Av. Vital Brazil, 1500, São Paulo, SP 05503-900 Brazil; 5 ATOMWISE, 717 Market Street, Suite 800, San Francisco, CA 94103 USA; 6 Laboratorio de Bacteriología, Escuela de Medicina, Universidad de Valparaíso, Valparaíso, Chile

**Keywords:** *Neisseria gonorrhoeae*, Artificial intelligence, Peptidoglycan, Bactericidal, Computational modelling

## Abstract

**Background:**

*Neisseria gonorrhoeae* (Ng) causes the sexually transmitted disease gonorrhoea. There are no vaccines and infections are treated principally with antibiotics. However, gonococci rapidly develop resistance to every antibiotic class used and there is a need for developing new antimicrobial treatments. In this study we focused on two gonococcal enzymes as potential antimicrobial targets, namely the serine protease L,D-carboxypeptidase LdcA (NgO1274/NEIS1546) and the lytic transglycosylase LtgD (NgO0626/NEIS1212). To identify compounds that could interact with these enzymes as potential antimicrobials, we used the AtomNet virtual high-throughput screening technology. We then did a computational modelling study to examine the interactions of the most bioactive compounds with their target enzymes. The identified compounds were tested against gonococci to determine minimum inhibitory and bactericidal concentrations (MIC/MBC), specificity, and compound toxicity in vitro.

**Results:**

AtomNet identified 74 compounds that could potentially interact with Ng-LdcA and 84 compounds that could potentially interact with Ng-LtgD. Through MIC and MBC assays, we selected the three best performing compounds for both enzymes. Compound 16 was the most active against Ng-LdcA, with a MIC50 value < 1.56 µM and MBC50/90 values between 0.195 and 0.39 µM. In general, the Ng-LdcA compounds showed higher activity than the compounds directed against Ng-LtgD, of which compound 45 had MIC50 values of 1.56–3.125 µM and MBC50/90 values between 3.125 and 6.25 µM. The compounds were specific for gonococci and did not kill other bacteria. They were also non-toxic for human conjunctival epithelial cells as judged by a resazurin assay. To support our biological data, in-depth computational modelling study detailed the interactions of the compounds with their target enzymes. Protein models were generated in silico and validated, the active binding sites and amino acids involved elucidated, and the interactions of the compounds interacting with the enzymes visualised through molecular docking and Molecular Dynamics Simulations for 50 ns and Molecular Mechanics Poisson-Boltzmann Surface Area (MM-PBSA).

**Conclusions:**

We have identified bioactive compounds that appear to target the *N. gonorrhoeae* LdcA and LtgD enzymes. By using a reductionist approach involving biological and computational data, we propose that compound Ng-LdcA-16 and Ng-LtgD-45 are promising anti-gonococcal compounds for further development.

**Supplementary Information:**

The online version contains supplementary material available at 10.1186/s40659-024-00543-9.

## Background

*Neisseria gonorrhoeae* (NgO) is the causative agent of the sexually transmitted infection gonorrhoea. NgO infects principally the mucosal epithelium of the human lower reproductive tracts. In men, infection of the urethra causes urethritis and painful discharge, and in women, infection of the endo/ectocervix leads to a mucopurulent cervicitis. However, in approximately 10–25% of untreated women, gonococci can ascend into the upper reproductive tract and cause pelvic inflammatory disease, which can leave patients with long-term and/or permanent chronic pelvic pain, fallopian tube damage, endometritis, ectopic pregnancy, and infertility [1]. Furthermore, gonococci can also infect other body sites and cause rare, complicated Disseminated Gonococcal Infection (DGI) [2]. Co-infection with other sexually transmitted disease pathogens, *e.g.* HIV, *Treponema pallidum* (syphilis), *Trichomonas* and *Chlamydia*, is common and gonococci are known to increase HIV transmission and infection [3].

Worldwide, there are ~ 87 million cases of gonococcal infection reported annually by the World Health Organisation (WHO) [4], with the majority in the least developed and low-to-middle income countries, and this number is probably an underestimate due to unreported asymptomatic infection. Gonococcal infection presents a high burden for treatment and support by public health and social services and in the absence of a prophylactic vaccine [5], control depends entirely on avoiding pathogen transmission through barrier protection and the use of antibiotics [6]. Both the Centers for Disease Control and Prevention (CDC) [7, 8] and the British Association for Sexual Health and HIV [9] now only recommend intramuscular ceftriaxone for treating typical, uncomplicated gonorrhoea (infection of the cervix, urethra or rectum), given the rise in resistance to azithromycin. For complicated DGI, the CDC recommends a variety of antibiotics including ceftriaxone, azithromycin and cefotaxime, depending on the clinical presentation and review of antimicrobial susceptibility testing data [10].

The gonococcus has a remarkable ability to use many different molecular pathways to rapidly develop resistance to every antibiotic class. This includes resistance to sulphonamides first introduced in the 1930s to treat gonorrhoea, to penicillins introduced in the 1940s, to tetracycline introduced in the 1950s and to the quinolone ciprofloxacin, introduced in the 1980s and abandoned by the mid-2000s. Over the past decade, there has been an increase in the number of reports of globally circulating multi-antibiotic resistant (MAR) gonococci that are difficult to treat, with the first reports in 2016–2018 in the UK of failures to treat pharyngeal gonorrhoea with ceftriaxone monotherapy and dual ceftriaxone-azithromycin therapy [11, 12]. The WHO has classified *N. gonorrhoeae* as a ‘High Priority’ MAR pathogen and currently, only solithromycin, zoliflodacin, SMT-571 and gepotidacin are new antibiotics in clinical evaluation for treating uncomplicated gonorrhoea [13–16]. Worryingly, their limitations will probably make them redundant through known resistance mechanisms, and a randomised phase 3 non-inferiority trial with solithromycin showed that it was not a suitable alternative to ceftriaxone [14]. There has been a resurgence of interest in repurposing abandoned antibiotics and in developing new antibiotics/antimicrobials for treating gonorrhoea, but developing any new antibiotic is a hugely expensive exercise, with (1) median development costs for a new antibiotic exceeding $1 billion; and (2) post-approval work and compound manufacture costs of ~ $350 m during the first 10 market years [17].

In the current study, we focused on two gonococcal enzymes as potential antimicrobial targets, namely the serine protease L,D-carboxypeptidase LdcA (NgO1274/NEIS1546) and the lytic transglycosylase LtgD (NgO0626/NEIS1212). *N. gonorrhoeae* (Ng)-LdcA was identified as the enzyme that converts cell wall tetrapeptide-stem peptidoglycan to release tripeptide-stem peptidoglycan [18]. The enzyme also has endopeptidase activity. Peptidoglycan fragments are recognised by intracellular NOD1 and NOD2 in mammalian cells, and Ng-LdcA plays a role in inflammation by modifying liberated peptidoglycan fragments into NOD1 agonists, with a secondary effect of generating NOD2 agonists via decreasing the supply of tripeptide substrate to make dipeptide [18]. *N. gonorrhoeae* (Ng)-LtgD was identified as the enzyme responsible for releasing peptidoglycan monomers from gonococci [19, 20]. Ng-LtgD in collusion with Ng-LtgA has been reported to protect gonococci from neutrophil killing by contributing to envelope integrity and limiting gonococcal exposure to antimicrobial molecules such as lysozyme [21]. Both Ng-LtgD and Ng-LtgA may also play a role in immunosuppression, since it has been suggested that both enzymes reduce recognition of gonococci by Toll-like Receptor 2 and NOD2 [22].

To identify potential compounds that could interact with these enzymes as potential antimicrobials, we used the AtomNet virtual high-throughput screening (HTS) technology [23–26]. Recently, the AtomNet convolutional neural network has been used in the largest and most diverse virtual HTS program to date, in 318 individual projects across 482 academic labs and screening centres from 257 different academic institutions in 30 countries. In this program, AtomNet successfully found novel hits across every major therapeutic area and protein class, and the selected molecules were novel drug-like scaffolds rather than just minor modifications to known bioactive compounds [27]. In the current study, we followed up the AtomNet identification of hit compounds against Ng-LtgD and Ng-LtgA with i) a computational modelling study to examine the deeper interactions of the most bioactive compounds with these target enzymes, and ii) testing of the compounds for functional bactericidal activity against gonococci.

## Methods

### AtomNet identification of compounds

Homology models were generated for the enzymes Ng-LdcA and Ng-LtgD to be used in the identification of small molecule inhibitors. The templates used for Ng-LdcA and Ng-LtgD were *E. coli* 5Z01 (1.75 Å) [28] with a sequence identity of 41% and *E.coli* 1D0K (2.02 Å) [29] with a sequence identity of 37%, respectively. The binding site of interest for LdcA was defined by chain A residues: D (Asp)73, R (Arg)76, R (Arg)133, G (Gly)134, G (Gly)135, F (Phe)164, S (Ser)165, D (Asp)166, M (Met)187, S (Ser)190, N (Asn)236, S (Ser)238, V (Val)239, D (Asn)261, V (Val)262, E (Glu)264, R (Arg)294, and H (His)331. The binding site of interest for LtgD was defined by chain A residues: M (Met)101, I (Ile)157, E (Glu)158, N (Asn)160, N (Asn)164, R (Arg)184, Y (Tyr)213, A (Ala)214, Q (Gln)221, F (Phe)222, M (Met)223, S (Ser)226, Y (Tyr)256, Q (Gln)340, Y (Tyr)341, N (Asn)342, H (His)343, and Y (Tyr)347.

The virtual high-throughput screening was performed using the AtomNet technology [23–27]. Briefly, the virtual screen was performed with the homology models generated for Ng-LdcA and Ng-LtgD at the binding sites of interest against approximately 8 million compounds in the MCULE v20191203 (https://mcule.com/) library. The compounds were filtered based on Lipinski’s rules, physiochemical properties, unwanted groups, and AtomNet score followed with clustering to ensure chemical diversity [30]. This then resulted in 74 compounds for Ng-LdcA and 84 compounds for Ng-LtgD, where two samples of dimethyl sulfoxide were included as an internal negative control, that were experimentally analysed as described below. Compounds were provided by Atomwise (purchased from Enamine) at a concentration of 10 mM in DMSO. Additional batches of Compound Ng-LdcA-16 and Ng-LtgD-45 were purchased from Chemspace (https://chem-space.com/) and Enamine (https://enamine.net/).

### Computational modelling of *Neisseria* gonorrhoeae LdcA and LtgD and analyses of their interactions with compounds

#### Protein modelling

For modelling purpose, the protein sequences of our *Neisseria gonorrhoeae* target proteins, a serine-protease L,D-carboxypeptidase, NGO1274/NEIS1546 (Ng-LdcA) with 393 amino acids and a lytic transglycosylase LtgD, NGO0626/NEIS1212 (Ng-LtgD) with 363 amino acids, were retrieved from the NCBI with Uniprot accession numbers A0A1D3GEK4 and A0A066SSQ2, respectively. Using PDB structural BLAST, multiple sequence alignment, proteins with high degrees of query coverage, and homology, we selected 5Z01 (Native *Escherichia coli* L,D-carboxypeptidase A, LdcA) and 1D0K (Native *Escherichia coli* Lytic Transglycosylase D, LtgD) as the structural templates. Template based protein models were generated using the AlphaFold artificial intelligence program [31]. Subsequently, the generated models were validated to assess model accuracy using PDBsum [32] and PROCHECK servers [33].

#### Model validation

The Ng-LdcA and Ng-LtgD models were superimposed with their respective template structures using PyMol (PyMol molecular graphics system, version 2.4.0 [34] molecular visualization system) to calculate the root mean square deviation (RMSD) of coordinates between the homology model and the template structures. Different web servers like SWISS-MODEL, Phyre2, Bhageerath-H [35–37] were used to validate and compare our generated models. Using the PROCHECK server [33], the accuracy of both the predicted models and their stereo-chemical properties were assessed using the Ramachandran plots and the overall goodness factor (G-factor). In addition, the models were analysed by PDBsum online server [32].

#### Identification of binding sites

The active site for binding of ligands was predicted using DeepFold (a deep learning based server which works on spatial restraint-guided structure prediction) [38].

#### Molecular docking studies

The six shortlisted compounds were used to perform the docking studies. The small molecule inhibitor compounds were characterized and energy minimized using conjugate gradient and steepest descent methods and docked with the modelled proteins i.e. Ng-LdcA and Ng-LtgD using AutoDock Vina [39], an open-source program for doing molecular docking. The docking scores were obtained in terms of a combined scoring function, which calculates the affinity, or fitness, of protein–ligand binding by summing up the contributions of several individual terms.

#### Molecular dynamics simulations (MDS)

MDS studies were carried out to gain a better understanding of the stability and dynamical properties of protein bound to the small molecules. The Ng-LdcA and Ng-LtgD models complexed with the identified novel small molecule inhibitors was subjected to MDS for 50 ns using GROMACS 2020.1 [40] package. In addition, a 50 ns MD run for the control compound was run. Gromos96 43a1force field [41] was used to generate the protein force fields, while Prodrg server [42] was used to generate the ligand topology force fields for the reference and potential inhibitor compounds. Briefly, these complexes were immersed in a cubic box of extended simple point-charge (SPC) water molecules. The complexes were neutralized by adding Na^+^ ions and Cl^−^ ions. To relieve the short-range bad contacts, energy minimization was done using the steepest descent method for 5000 steps. Initially, the position-restrained simulations were carried out at 298 K for 100 ps. The three complexes were subjected to a 50 ns MDS production run and the control at 298 K temperature and 1 bar pressure, using a 0.002 ps time step. The Parrinello–Rahman method [43] was used to control the pressure and a V-rescale thermostat was used to maintain the temperature. The long-range electrostatic forces were handled using the Particle Mesh Ewald (PME) method [44] with a real-space cut-off of 10 Å, PME order of six, and a relative tolerance between long and short-range energies of 10^– 6^ kcal/mol. Short-range interactions were evaluated using a neighbour list of 10 Å updated every 10 steps, while Lennard–Jones (LJ) interactions and the real-space electrostatic interactions were truncated at 9 Å. Hydrogen bonds were constrained using the LINCS algorithm [45]. The final models in all the complexes were obtained by averaging the snapshots from the trajectory generated by MD simulations after the structure stabilization was achieved. To study the effect of inhibitor binding on the stability of Ng-LdcA and Ng-LtgD, we also performed the MDS studies of protein bound with the substrate (i.e. Ng-LdcA and Ng-LtgD in unbound state) under similar conditions. To study the conformational variations in the structures of Ng-LdcA-substrate and Ng-LdcA-inhibitor complexes and Ng-LtgD-substrate and Ng-LtgD-inhibitor complexes, the Root-Mean-Square Deviations (RMSD) of the Ca atoms were calculated. The convergence of MDS was analysed in terms of the potential energy, RMSD, SASA, H-bond analysis, and Radius of Gyration (RoG) [46].

#### Molecular Mechanics Poisson-Boltzmann Surface Area (MM-PBSA)

To gain more insight into the structural dynamics of the protein–ligand complex and to further validate the MDS findings, free energies of docked complexes from the lead compounds along with the control were calculated using Molecular Mechanics Poisson-Boltzmann Surface Area (MM-PBSA) using the g_mmpbsa tool. From the last 10 ns of each MD run (i.e. from 40 to 50 ns), MM-PBSA calculations were done with all the default parameters. Also, the binding energies of these protein–ligand complexes were enumerated using the following equation as reported earlier [47]. The receptor’s binding free energy with its ligand in a solvent can be expressed as Eq. (1)

1 $${\mathbf{DG}}_{{{\mathbf{bind}}{\mathbf{.pred}}}} = \Delta {\mathbf{Gcomplex}}{-} \, \left[ {{\mathbf{DG}}_{{{\mathbf{Rec}}}} + {\mathbf{ DG}}_{{{\mathbf{lig}}}} } \right]$$

where DG_complex_ is receptor-ligand, total free energy and DG_Rec_ and DG_lig_ are isolated total free energies of receptor and ligand, respectively in a solvent. Furthermore, each individual entity’s free energy can be provided by Eq. (2)

2 $${\mathbf{G}} \, = \, {\mathbf{E}}_{{{\mathbf{gas}}}} + \, {\mathbf{G}}_{{{\mathbf{sol}}}} {-} \, {\mathbf{TS}}$$

where G is the free energy for ligand, receptor or receptor ligand complex, E_gas_ is molecular mechanics (MM) potential energy in gaseous state and G_sol_ is the solvation free energy of the respective entity. TS represents entropic contributions, where T and S refer to temperature and entropy, respectively. Equation (3) calculates various interactions.

3 $${\mathbf{E}}_{{{\mathbf{gas}}}} = \, {\mathbf{E}}_{{{\mathbf{int}}}} + \, {\mathbf{E}}_{{{\mathbf{ele}}}} + \, {\mathbf{E}}_{{{\mathbf{vdw}}}}$$

where the E_gas_ includes both bonded interaction (E_int_) as well as non-bonded interaction (E_ele_ + E_vdw_) terms based on MM force field parameters. The solvation free energy was calculated by Eqs. (4) and (5).

4 $${\mathbf{G}}_{{{\mathbf{sol}}}} = \, {\mathbf{G}}_{{{\mathbf{PB}}({\mathbf{GB}})}} + \, {\mathbf{G}}_{{{\mathbf{sol}} - {\mathbf{np}}}}$$

5 $${\mathbf{G}}_{{{\mathbf{sol}} - {\mathbf{np}}}} = \, {\mathbf{\gamma SAS}}$$

In Eq. (4), G_sol_ is solvation free energy calculated in terms of electrostatic (G_PB(GB)_) and non-electrostatic (G_sol-np_) contributions, whereas in Eq. (5), SAS represents solvent accessible surface area of the model.

### Bacteria

*Neisseria gonorrhoeae* strain P9-17, a 1B-26 serovar isolate (ND: P1.18–10,43: F1-26: ST-1926, Pil^+^ Opa_b_^+^; PubMLST ID 36675) was originally isolated from a patient with gonococcal prostatitis [48]. *Neisseria meningitidis* strain MC58 (B:15:P1.7,16b, PubMLST ID 240) was isolated from an outbreak of meningococcal infections that occurred in Stroud, Gloucestershire in the mid-1980’s [49]. *Neisseria lactamica* strain Y92-1009 (sequence type 3493, clonal complex [CC] 613, PubMLST ID 4945) was produced by the Current Good Manufacturing Practices pharmaceutical manufacturing facilities at Public Health England (Porton Down, United Kingdom). *N. gonorrhoeae* isolates assembled by the Centers for Disease Control and Prevention (CDC) in collaboration with the Food and Drug Administration (FDA) were also used, namely AR-0166, AR-0173, AR-0174, AR-0190 and AR-0194 (https://www.cdc.gov/drugresistance/resistance-bank/currently-available.html), which reported resistance to ceftriaxone and azithromycin. All *Neisseria spp* were grown on supplemented GC-agar plates [50] incubated at 37°C in an atmosphere containing 5% (v/v) CO_2_. *Pseudomonas aeruginosa* strain PAO-1 (Holloway1C Stanier131) was obtained from the National Collection of Industrial, Food and Marine Bacteria, UK and grown on Luria–Bertani agar (Oxoid, Basingstoke, UK) at 37 °C. *Staphylococcus aureus* strains NCTC8325.4, 6517 and 29213, *S. capitis* strain 09E395 and *S. epidermidis* strain 12228 were all obtained from LGC (Middlesex, UK) and were grown on Luria–Bertani agar (Oxoid, Basingstoke, UK) at 37 °C. *Lactobacillus gasseri* was grown on Tryptic Soy Agar (Oxoid) at 37 °C with 5% (v/v) CO_2_ [51].

### Standard GC broth microdilution assay for determining minimum inhibitory concentration (MIC) and minimum bactericidal concentration (MBC)

Initially, all compounds were screened for anti-gonococcal activity at a dose of 50 μM using a broth microdilution assay that followed the Clinical and Laboratory Standards Institute guidelines (M07-A8 and M26-A) with some minor modifications [52]. Gonococci were grown overnight and cultured the following day for 6 h to provide growth in the exponential phase. Suspensions of gonococci were measured spectrophotometrically using a Nanodrop and diluted to a concentration of 5 × 10^5^ colony forming units/ml in supplemented GC broth. To the wells of sterile 96 well plates (Nunc) were added 190 μl of bacterial suspension, i.e. final concentration of ~ 10^5^ CFU/well, and 10 μl of each compound in triplicate wells to a final concentration of 50 μM in the well. Negative controls were GC broth alone and GC broth with 10 μl of DMSO. The positive control was ceftriaxone. The plates were incubated for 24 h at 37 °C with 5% (v/v) CO_2_ and absorbance measured with a SpectraMax iD3 plate reader (Molecular Devices, San Jose, CA, USA) at λ 595 nm. Compounds that induced a reduction in absorbance of > 50% at 50 μM, when compared to bacteria without treatment, were chosen for determining MIC values. These compounds were then serially diluted in two-fold from 50 μM and tested against the bacteria, and the MIC value was interpreted as the concentration of a test compound that inhibited the growth of gonococci following overnight incubation and was determined at the 50% (MIC50) and > 90% levels (MIC90). MIC values were also obtained for ceftriaxone similarly. The active compounds also were screened against other *Neisseria spp* and other bacteria (*P. aeruginosa, Staphylococcus spp., L. gasseri)* similarly.

From the same 96 well plates used to determine MIC values, samples were plated onto agar and incubated overnight at 37 °C with 5% (v/v) CO_2_ and viable bacteria counted. The MBC50 and MBC90 were recorded as the concentrations that reduced growth of any visible colonies after culture, compared to the untreated controls, by 50% and > 90% respectively.

### Modified bactericidal assay for compound Ng-LtgD-45

The method described by Lucio et al. and Santana et al. was used to test the activity of Ng-LtgD-45 against gonococci [53, 54]. Briefly, bacteria were grown from frozen stock as described above to exponential phase and 190 µL of bacterial suspension in Dulbecco’s phosphate buffered saline (PBSB) containing ~ 10^5^ CFU/well was added to wells of a 96-well plate in triplicate. Next, different doses of Ng-LtgD were added to each well in a volume of 10 µL, starting from an initial concentration in the well of 50 µM. Negative controls were bacteria in PBSB without treatment and the positive control was ceftriaxone. The plate was incubated for 1 h at 37 °C in a humidified atmosphere with 5% (v/v) CO_2_, and viable counts were then made as described above. The MBC50 and MBC90 were recorded as the concentrations that reduced growth of any visible colonies after culture, compared to the untreated controls, by 50% and > 90% respectively.

### Time-to-kill assay

*N. gonorrhoeae* strain P9-17 was treated with a test concentration of 50 μM of compound or ceftriaxone using a standard MBC protocol, and bacteria were sampled at 0, 1, 3, 6, 9 and 24 h by viable counting. Control was untreated bacteria alone.

### Resazurin cytotoxicity assay

Human Chang conjunctival epithelial cells (European Type Culture Collection, Porton Down, United Kingdom) were cultured in the wells of sterile 96-well cell culture plates (Nunc) at 37 °C in Dulbecco’s Modified Eagle’s Medium supplemented with Glutamax-1 and sodium pyruvate (DMEM) (Lonza, United Kingdom) and 10% (v/v) decomplemented fetal calf serum (dFCS) (Lonza). Cells were cultured in a humidified atmosphere at 37 °C with 5% (v/v) CO_2_. Prior to treatment, the medium was removed, and the cells washed to remove any dead cells and fresh medium added (190 μl/well). Next, 10 μl of test compound was added per well to a final concentration of 50 μM. Lysis solution was added as a positive control and ceftriaxone was also tested at 50 μM. The plates were incubated for 18 h at 37 °C with 5% (v/v) CO_2_ and then 20 μl of resazurin was added to each well. The plate was incubated for a further 4 h and the absorbance read at λ 570 nm and λ 595 nm for background correction on a SpectraMax iD3 plate reader. The plate was incubated for a further 20 h and absorbance measured again.

## Results

### Initial screening of compounds identified by AtomNet in MIC and MBC experiments.

**Fig. 1:**
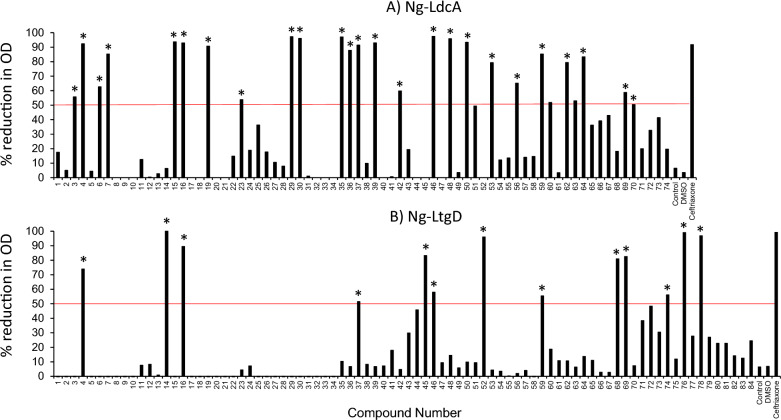
Screening of compounds in a standard Minimum Inhibitory Concentration (MIC) assay. Ng-LdcA (n = 74) and Ng-LtgD (n = 84) compounds were diluted and tested at a single concentration of 50 µM in wells containing ~ 10^5^ CFU of *N. gonorrhoeae* strain P9-17. Controls were bacteria alone, GC broth alone (control in the graphs), GC broth with DMSO (10µL/well) alone, and positive control was bacteria treated with ceftriaxone. DMSO alone has no effect on bacterial growth. Optical density was measured after 24 h incubation. The data are shown as the % reduction in optical density compared to the control bacteria alone. Data are from a representative experiment done twice. The red lines denote the 50% cut offline for selecting compounds for further analyses

The AtomNet technology identified 74 compounds that could potentially interact with Ng-LdcA and 84 compounds that could potentially interact with Ng-LtgD. Initially, all the compounds were screened at a concentration of 50 µM in the standard broth microdilution assay against *N. gonorrhoeae* strain P9-17 (Fig. 1). We identified 26 compounds for Ng-LdcA (Fig. 1A) and 13 compounds for Ng-LtgD (Fig. 1B) that reduced the optical density readings by greater than 50% and these were chosen for further study. These compounds were then serially diluted in the broth microdilution assay and tested against strain P9-17 to determine the MIC50/90 values, shown in Supplementary Table 1 for Ng-LdcA and Supplementary Table 2 for Ng-LtgD. The individual MIC titration curves are shown in Supplementary Fig. 1 for the Ng-LdcA compounds and in Supplementary Fig. 2 for the Ng-LtgD compounds. Next, the MBC50/90 values were determined for only the compounds with the lowest MIC values, i.e. those most likely to show highest bactericidal effects (highlighted in Supplementary Tables 1, 2; Supplementary Fig. 3). This reductionist screening led to the final selection of the most active compounds, with three Ng-LdcA compounds and three Ng-LtgD compounds showing the highest activities against P9-17: the structures and properties of these compounds are summarised in Table 1. Compound 16 was the most active against Ng-LdcA, with a MIC50 value < 1.56 µM, MIC > 90 values of 3.125–6.25 µM and MBC50/90 values between 0.195 and 0.39 µM. In general, the Ng-LdcA compounds showed higher activity than the compounds directed against Ng-LtgD, of which compound 45 had MIC50 values of 1.56–3.125 µM, MIC > 90 values of 6.25–12.5 µM and MBC50/90 values between 3.125 and 6.25 µM (Table 1).

**Table 1:**
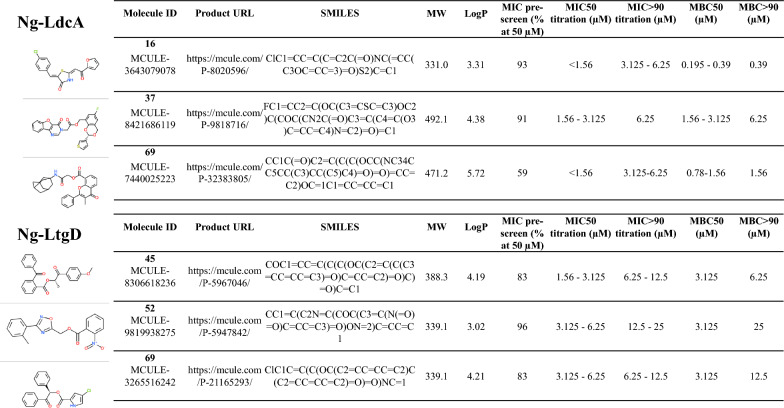
Summary of the structures, properties and MIC and MBC values for the selected Ng-LdcA and Ng-LtgD compounds

MW, molecular weight; SMILES, simplified molecular-input line-entry system; LogP, Log value of the partition coefficient; MIC, Minimum Inhibitory Concentration; MBC, Minimum Bactericidal Concentration

### Computational modelling of Ng-LdcA and Ng-LtgD and analyses of their interactions with the hit compounds

Having identified the three Ng-LdcA (-16, -37 and -69) and three Ng-LtgD (-45, -52 and -69) compounds from the initial biological screen, we then did a deep computational study to model their interactions with their target enzymes.

#### Protein modelling and validation

In the absence of crystal structures, homology modelling has become the primary method for obtaining a three-dimensional (3D) representation of protein targets in recent years. Since no crystal structure of Ng-LdcA and Ng-LtgD was available, it was determined using homology modelling with AlphaFold. From the structural PDB BLAST results, the Ng-LdcA sequence showed the best alignment with the native *Escherichia coli* L,D-carboxypeptidase A, LdcA (PDB Id: 5Z01) [28] with a query coverage of 99% and sequence similarity of 41%. The Ng-LtgD sequence showed the best alignment with the native *Escherichia coli* Lytic transglycosylase D, LtgD (PDB Id: 1D0K) [29] with a query coverage of 88% and sequence similarity of 37%. These sequence similarity findings agreed with the initial Atomwise models. The observed gaps between the template and query proteins were found to be 1%. The remaining 11% of the sequence, which corresponded to regions at the C- terminal of the Ng-LtgD protein, was not covered by the alignment and was excluded from the final generated model of our protein. This portion of the sequence, including the C-terminal region (MEKRKILPLAICLAALSACTAMEARTPRANEAQAPRADEMKK ESRPAFDAAAVPVSDSGFAAN), was excluded from the final model as it did not include residues relevant to the catalytic domain or active site. The sequence alignment, highlighting the modelled regions and indicating the excluded parts, is provided in Supplementary Fig. 4.

**Fig. 2:**
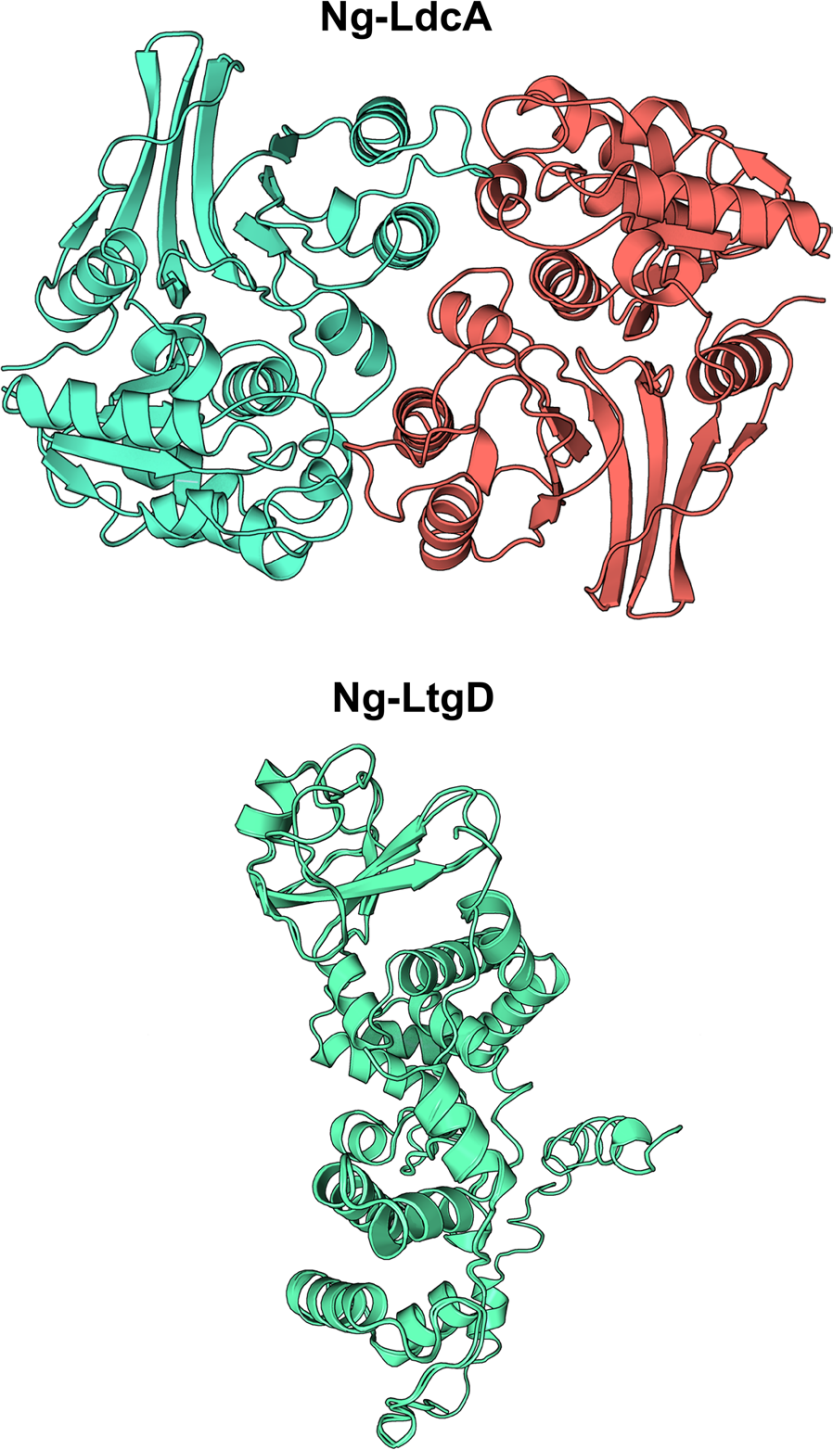
Homology-based models of Ng-LdcA and Ng-LtgD. The *E. coli* 5Z01 template-based model of Ng-LdcA and the *E. coli* 1D0K template-based model of Ng-LtgD were generated by AlphaFold. The Ng-LdcA homo-dimeric protein consists of two identical chains (Chain A in green and Chain B in red), whereas Ng-LtgD is a monomer (in green)

The 3D structure of Ng-LdcA was modelled based on the sequence alignment with *E. coli*-LdcA PDB Id: 5Z01 (Fig. 2) and the 3D structure of Ng-LtgD was modelled based on the sequence alignment with *E. coli*-LtgD PDB Id: 1D0K (Fig. 2). The modelled structure for Ng-LdcA was superimposed on the 5Z01 template structure and the RMSD value was calculated at 1.70 Å and the modelled structure for Ng-LtgD was superimposed on the 1D0K template structure and the RMSD value was calculated at 1.20 Å. These calculations indicated significant backbone similarity between the modelled protein and the template structure. Both the modelled structures were then validated with the PDBsum server and the Ramachandran plot (Supplementary Fig. 5), which revealed for Ng-LdcA that 90% of residues were in the allowed region, 9.6% in the favoured region and 0.4% was present in disallowed regions, whereas for Ng-LtgD, 81.7% of residues were in the allowed region, 14% in the favoured region, 2.6% in generously allowed region and 1.7% was present in disallowed regions. These values suggested good overall stereo-chemical quality and stability of the models. ERRAT analysis for the overall model quality factor, indicated that both generated models were of extremely high-quality and reliability with respect to 3D structure. The models’ backbone conformation, non-bonded interactions and energy scores were well within the range of the high-quality models. In the case of Ng-LdcA, two homologous chains A and B constituted the dimeric protein, whereas Ng-LtgD was a monomer.

#### Active site analysis and validation

**Fig. 3:**
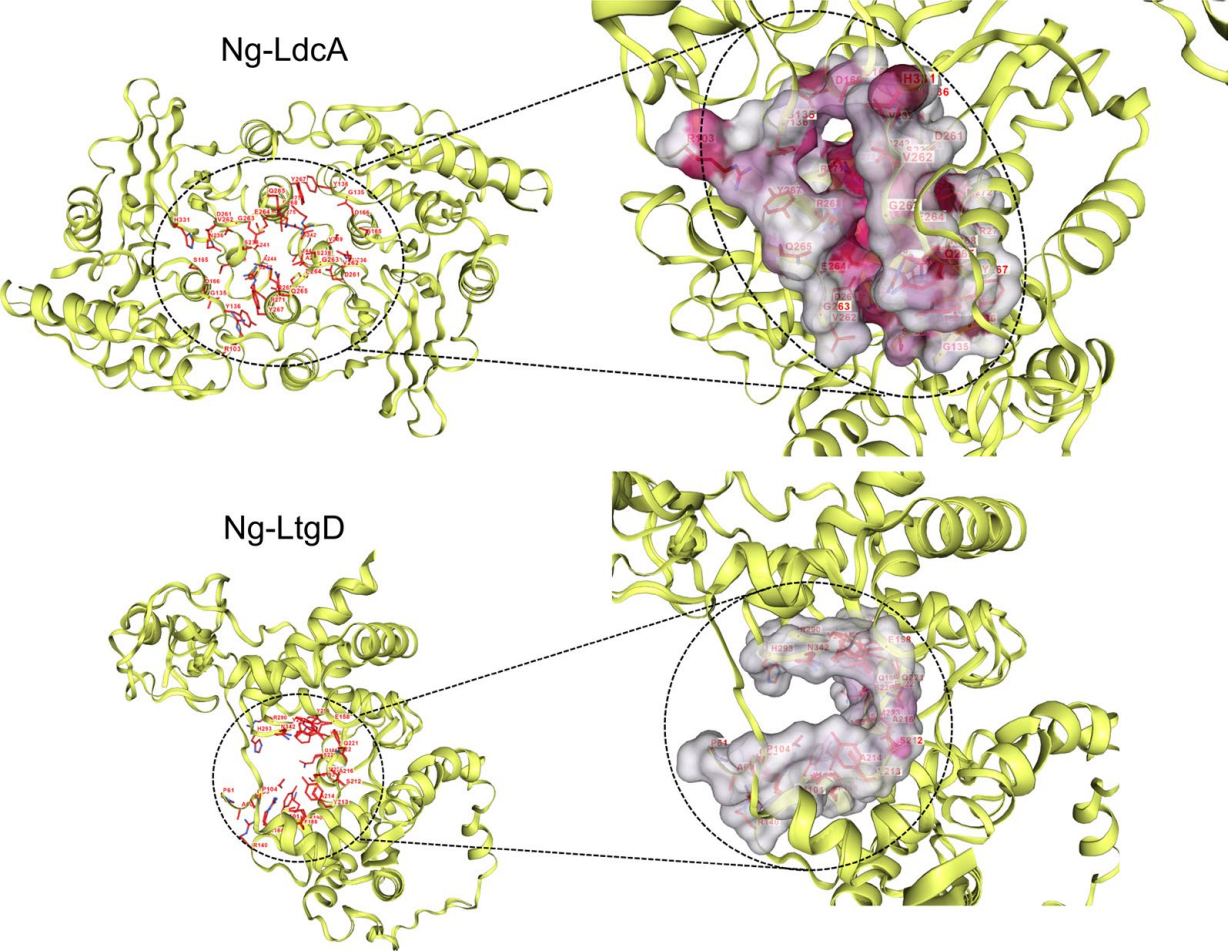
Identification of binding pockets with DeepFold. The binding pocket (active site) predicted by DeepFold is shown for Ng-LdcA and Ng-LtgD. The active site regions show the conserved amino acid residues predicted to be involved in protein–ligand binding and conserving the architecture of the binding cavity. See text for description of the amino acids involved

The identification of potential binding site(s) for the ligands provides an insight into the active site regions on the proteins and gives an idea of the interacting residues as well as the crucial interactions between the proteins and ligands. Identifying the binding site is essential for structure-based virtual screening of compound libraries. To access the binding cavity in the modelled Ng-LdcA protein, the Ng-LdcA moiety mostly contacted amino acid residues in the deep cleft between the enzyme’s two domains. As shown in Fig. 3 the residues from Chain A: Tyr267, Arg268, Arg271, Tyr302, Asp303, and from Chain B: Gly69, Phe70, Glu72, Arg103, Arg133, Gly135, Tyr136, Ser165, Asn236, Ser238, Val239, Asp261, Val262, Glu264, formed the main region of the binding site and were conserved across multiple *Neisseria* species and homologs in other bacteria. To access the binding cavity in the modelled Ng-LtgD protein, as shown in Fig. 3, the residues from the protein chain of Met101, Ile157, Glu158, Asn160, Asn164, Arg184, Tyr213, Ala214, Gln221, Phe222, Met223, Ser226, Tyr256, Gln340, Tyr341, Asn342, His343, Tyr347, formed the main region of the binding site and were similarly conserved. The conserved nature of active site residues across different species gives us confidence in the relative accuracy of both the models [55–57]. The identified active sites were confirmed with the help of previously reported literature for Ng-LdcA and Ng-LtgD protein complexes and validated by repeating the targeted docking and creating the binding cavity using the same residues.

#### Molecular docking

**Fig. 4:**
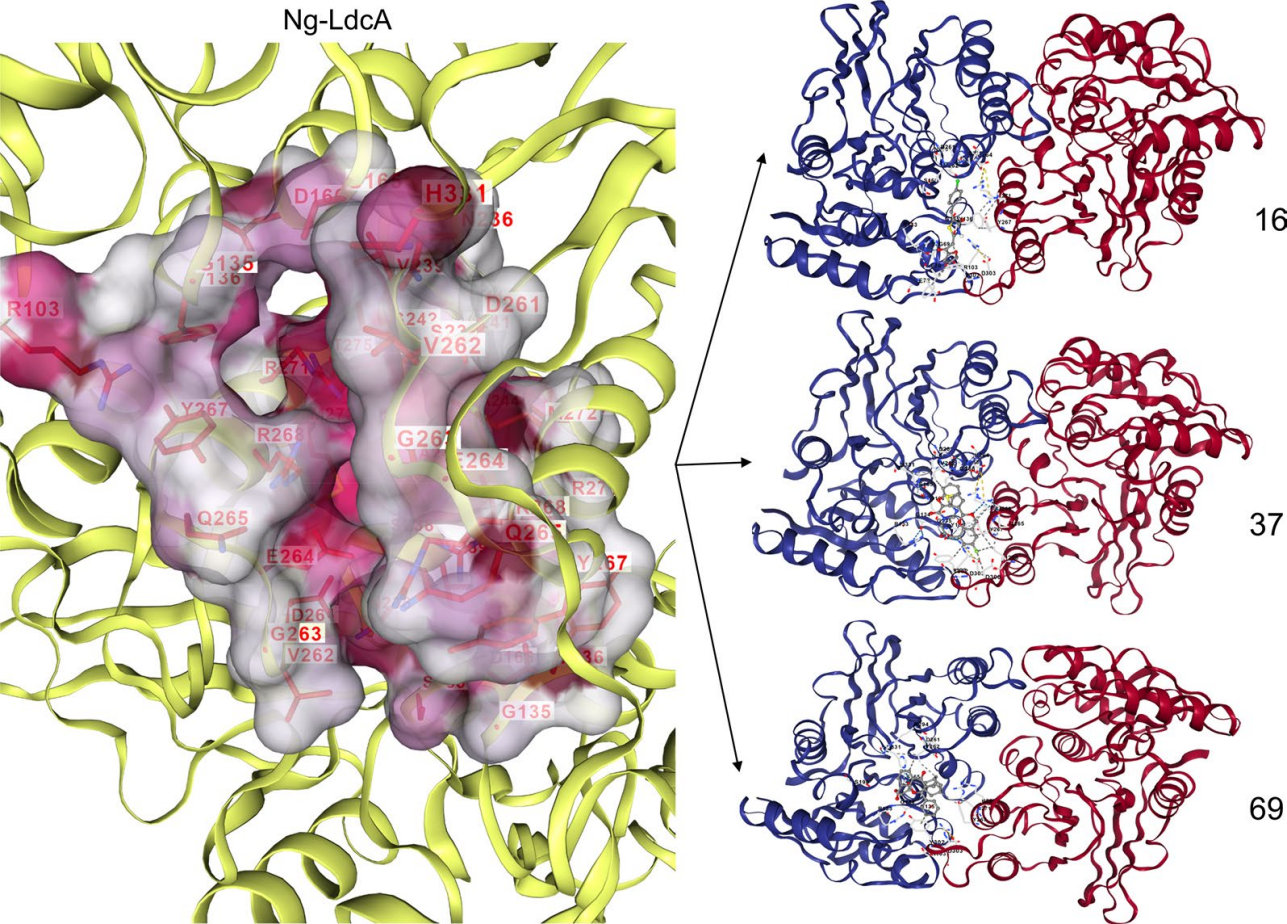
Molecular docking studies for Ng-LdcA. The left-hand exploded diagram shows the binding site at the surface of modelled protein for Ng-LdcA. On the right-hand side are images of compounds Ng-LdcA-16, -37 and -69 docked in the binding cavity. All the docked ligands exhibited good docking scores and retained all the conserved residues in the protein–ligand binding interaction

**Fig. 5:**
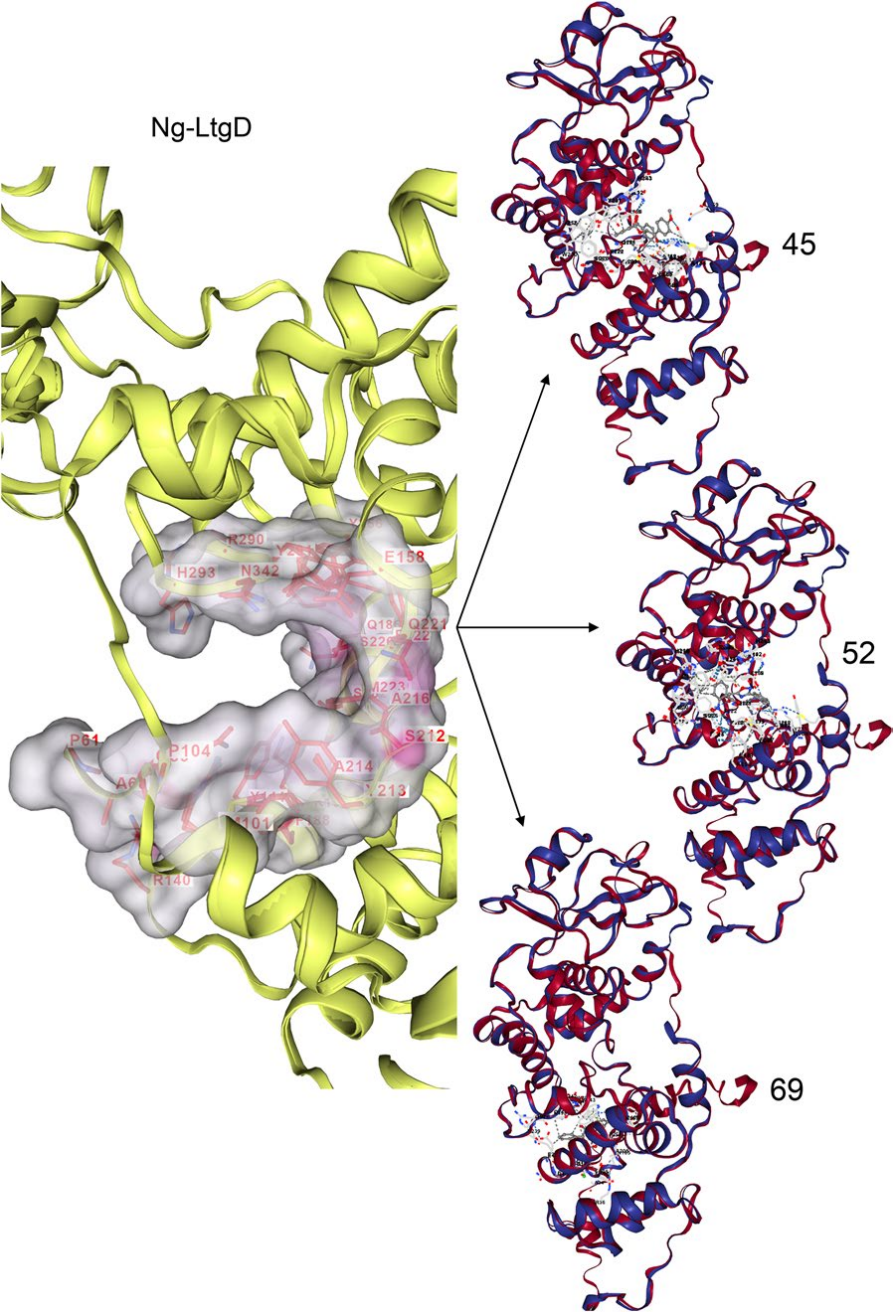
Molecular docking studies for Ng-LtgD. The left-hand exploded diagram shows the binding site at the surface of modelled protein for Ng-LtgD. On the right-hand side are images of compounds Ng-LtgD-45, -52 and -69 docked in the binding cavity. All the docked ligands exhibit good docking scores and retained all the conserved residues in the protein–ligand binding interaction

**Fig. 6:**
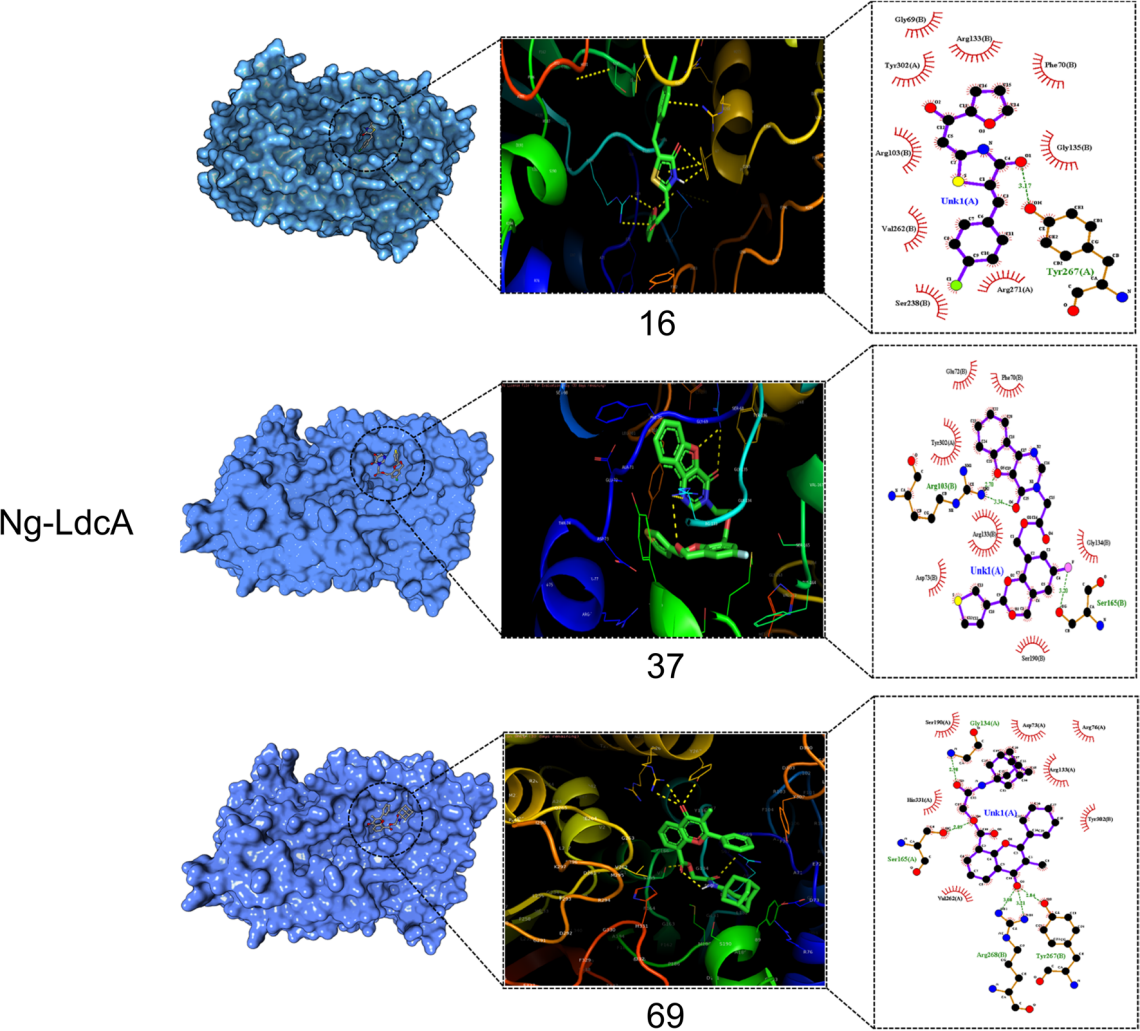
LigPlot^+^ 2D interaction diagrams for Ng-LdcA. The 2D interaction diagrams of the compounds docked with modelled Ng-LdcA proteins, plotted using LigPlot^+^. The interactions of all the molecules obtained by molecular docking are shown for compounds Ng-LdcA-16, -37 and -69

**Fig. 7:**
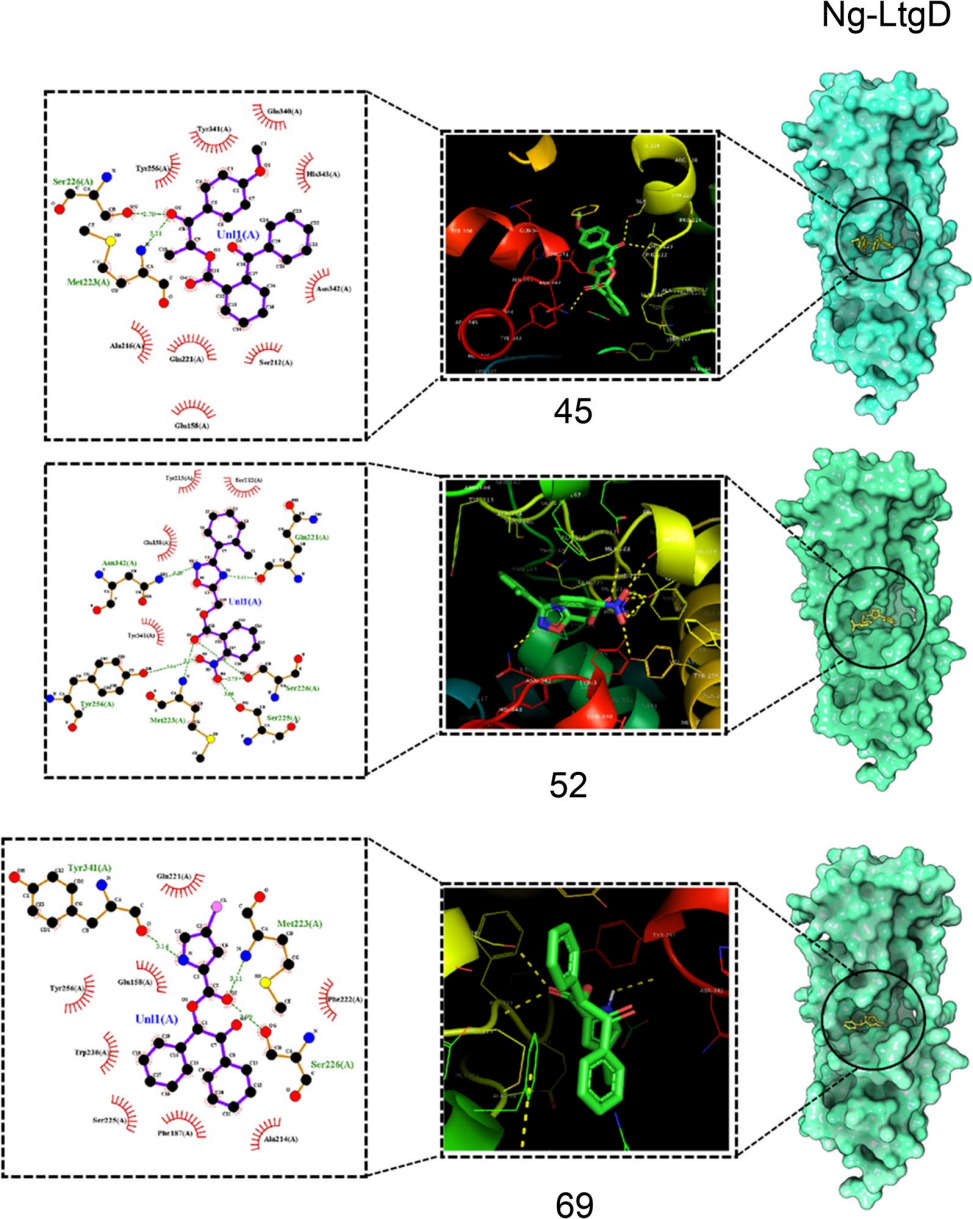
LigPlot^+^ 2D interaction diagrams for Ng-LtgD. The 2D interaction diagrams of the compounds docked with modelled Ng-LtgD proteins, plotted using LigPlot^+^. The interactions of all the molecules obtained by molecular docking are shown for compounds Ng-LtgD-45, -52 and -69

This was used to assess the binding ability of the three Ng-LdcA and three Ng-LtgD hit compounds to the target Ng-LdcA and Ng-LtgD proteins. The compounds were docked and modelled onto Ng-LdcA and Ng-LtgD using AutoDock Vina to evaluate their binding affinity and to investigate how precisely the ligands docked into the binding region at the protein surface. All the successfully docked compounds exhibited favourable binding interactions with Ng-LdcA and Ng-LtgD (Table 2) and the docked compounds are shown in Fig. 4 for Ng-LdcA and Fig. 5 for Ng-LtgD with their interactions and best docked pose selected based on the docking scores. These compounds showed good interactions with some of the substrate-binding amino acids, such as Gly69, Phe70, Glu72, Arg103, Arg133, Gly135, Tyr136, Ser165, Asn236, Ser238, Val239, Asp261, Val262, Glu264, Tyr267, Arg268, Arg271, Tyr302 and Asp303 for Ng-LdcA, and Met101, Ile157, Glu158, Asn160, Asn164, Arg184, Tyr213, Ala214, Gln221, Phe222, Met223, Ser226, Tyr256, Gln340, Tyr341, Asn342, His343 and Tyr347 for Ng-LtgD, and they fit well into the active site cavity of their respective proteins. The significant interactions of these compounds plotted by LigPlot^+^ are shown in Fig. 6 for Ng-LdcA and Fig. 7 for Ng-LtgD.

**Table 2:** Characteristics of hit compounds with their docking, free energy and binding affinity scores

Enzyme	Compound ID	Structure	Docking score (kcal/mol)	Estimated ΔG (kcal/mol)	Binding Affinity (kcal/mol)
Ng-LdcA	16 MCULE-3643079078		− 2509.91	− 8.06	5.3
Ng-LdcA	37 MCULE-8421686119		− 2488.46	− 7.82	5.6
Ng-LdcA	69 MCULE-7440025223		− 2456.21	− 7.51	4.5
Ng-LtgD	45 MCULE-8306618236		− 4207.40	− 11.24	6.3
Ng-LtgD	52 MCULE-9819938275		− 4229.70	− 10.49	7.1
Ng-LtgD	69 MCULE-3265516242		− 4247.16	− 10.37	6.9

#### Molecular dynamics simulations (MDS) of compounds

**Fig. 8:**
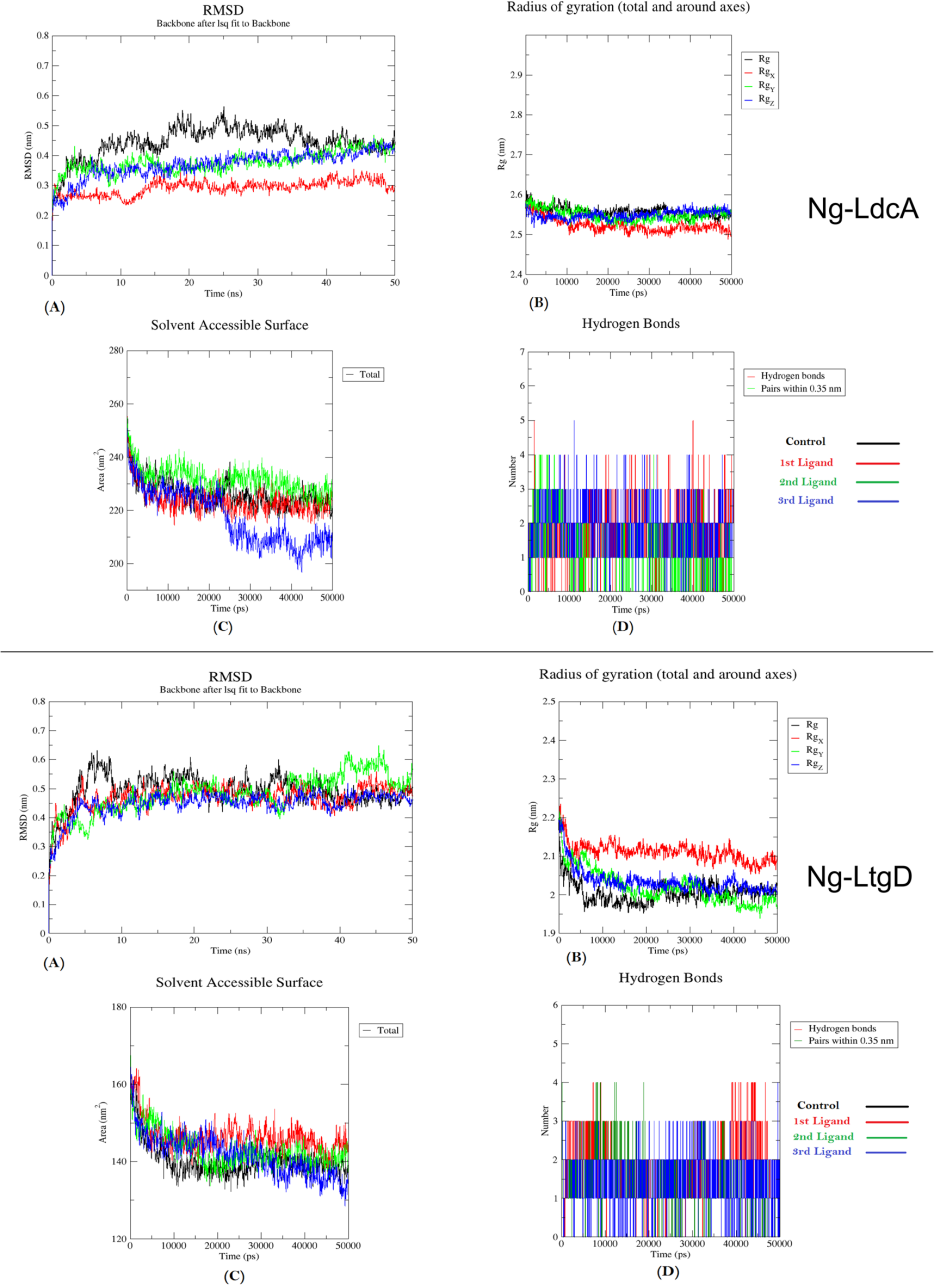
Plots to investigate the energy deviation, conformation stability and surface area accessible during simulation for Ng-LdcA and Ng-LtgD proteins and ligands in bound state with the protein. **A** represents RMSD, **B** represents radius of gyration, **C** represents Solvent Accessible Surface Area (SASA), and **D** represents number of hydrogen bonds. Black colour shows control, whereas red, green and blue colour shows 1st, 2nd and 3rd ligands respectively. Ng-LdcA 1st ligand = compound -16, 2nd = compound -37, 3rd = compound -69. Ng-LtgD 1st ligand = compound -45, 2nd = compound -52, 3rd = compound -69

To gain insights into the stability and dynamic properties of the protein–ligand complexes, explicit solvent MDS were done. MDS provided detailed insight into protein–ligand interactions in motion, contributing to their stable bound conformation and visualizing the effect of ligand binding on protein conformational changes. MDS for up to 50 ns were done for control (protein alone) as well as for the compounds docked with protein. The trajectories were also analysed for all the protein–ligand docked complexes after completion of the simulation run. The time evolution of the Root Means Square Deviation (RMSD) during MDS is used to monitor protein stability. The distributional probability of RMSD up to 50 ns trajectories is shown in Fig. 8. The mean RMSD values of Ng-LdcA-control complex, Ng-LdcA-16, Ng-LdcA-37 and Ng-LdcA-69 complex were ~ 0.2–0.4 nm, which shows the structural stability throughout the MD run [58, 59], where the RMSD values of Ng-LdcA-16 were found to be more stable (Fig. 8A, top panel). We also calculated the radius of gyration (Rg) value, a parameter directly associated with the overall conformational changes in the structure of the enzyme upon ligand binding, to further validate our results. It also revealed the stability, compactness, and folding behaviour of the structure. We calculated the Rg values of all the selected compounds and the reference complex to determine their compactness. The average Rg values for the Ng-LdcA-control complex, Ng-LdcA-16, Ng-LdcA-37 and Ng-LdcA-69 complex were below 2.6 nm, were stable after 15 ns implying increased compactness, and improved binding (Fig. 8B, top panel). In addition to RMSD and Rg, the number of hydrogen bonds generated between protein and ligand throughout the simulation duration was determined. Figure 8D (top panel) displays the graphs of these H-bonds formed between the protein and the corresponding ligand throughout the 50 ns simulation run. The average number of H-bonds formed between Ng-LdcA-16 is highest followed by Ng-LdcA-37. We have also analysed an important parameter, which is SASA (Solvent-Accessible Surface Area), which is an approximate surface area of a biomolecule that is accessible to a solvent with respect to the simulation time. Figure 8C (top panel) indicates that for Ng-LdcA-16 and Ng-LdcA-control complex the solvent accessible surface was optimally acquired and within the acceptable range [60, 61].

Similarly, in the case of Ng-LtgD, the distributional probability of RMSD up to 50 ns trajectories is shown in Fig. 8 (bottom panel). The mean RMSD values of Ng-LtgD-control complex, Ng-LtgD-45, Ng-LtgD-52 and Ng-LtgD-69 complex were originated from around 0.2 nm and ranges up-to 0.6 nm, which shows the structural stability throughout the MD run, where the RMSD values of Ng-LtgD-69 were found to be more stable (Fig. 8A, bottom panel). We also calculated the Rg values of all the selected compounds and the reference complex to determine their compactness. The average Rg values for the Ng-LtgD-control complex, Ng-LtgD-45, Ng-LtgD-52 and Ng-LtgD-69 complex were below 2.2 nm, were stable after 10 ns implying increased compactness, and improved binding (Fig. 8B, bottom panel). Figure 8D (bottom panel) displays the graphs of the H-bonds formed between the protein and the corresponding ligand throughout the 50 ns simulation run. The average number of H-bonds formed between Ng-LtgD-69 is highest followed by Ng-LtgD-52. In addition, Fig. 8C (bottom panel) indicates that for Ng-LtgD-69 and Ng-LtgD-control complex the SASA was optimally acquired and within the acceptable range (50–200 nm^2^) [60, 61].

#### Molecular mechanics Poisson-Boltzmann surface area (MM-PBSA)

**Fig. 9:**
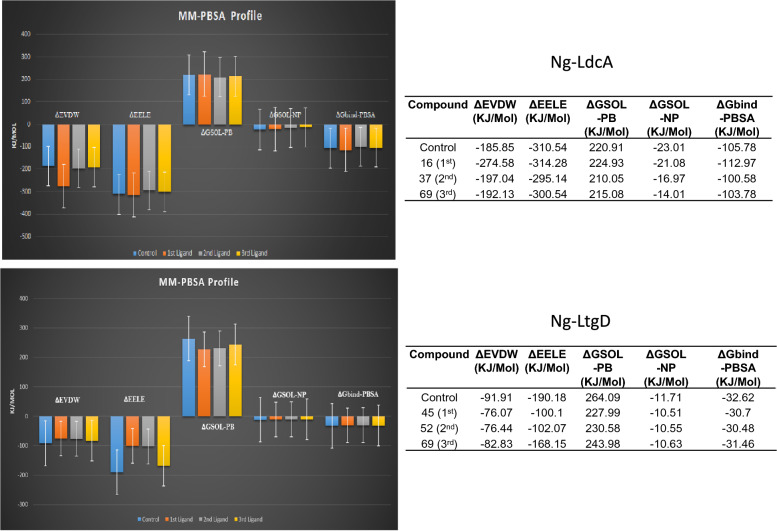
Binding free energy calculations using MM-PBSA tool for the potential small molecule inhibitor compounds along with the control. Colour coding is represented in the figure. Ng-LdcA 1st ligand = compound -16, 2nd = compound -37, 3rd = compound -69. Ng-LtgD 1st ligand = compound -45, 2nd = compound -52, 3rd = compound -69

Conformations from the last 10 ns MDS run were used to gain more insight into the structural dynamics of protein–ligand complexes. To validate the MDS findings, free energies of docked complexes from all the potential inhibitor small molecules along with control were calculated using MM-PBSA with the g_mmpbsa tool (Fig. 9). All the complexes exhibited negative binding energy in the MM-PBSA threshold. As shown, in case of Ng-LdcA, out of the three compounds, Ng-LdcA-16 exhibited a better net binding energy score which may be comparable with the binding energy of control. The binding energy score for Ng-LdcA-16 as depicted by MM-PBSA, exhibited better protein–ligand binding compared to two other compounds. Similarly, for Ng-LtgD, out of the three compounds, Ng-LtgD-69 exhibited a better net binding energy score, which may be comparable with the binding energy of control. The binding energy score for Ng-LtgD-69 depicted by MM-PBSA exhibited better protein–ligand binding, compared to the two other compounds.

### Cytotoxicity of the compounds for human cells in vitro

**Fig. 10:**
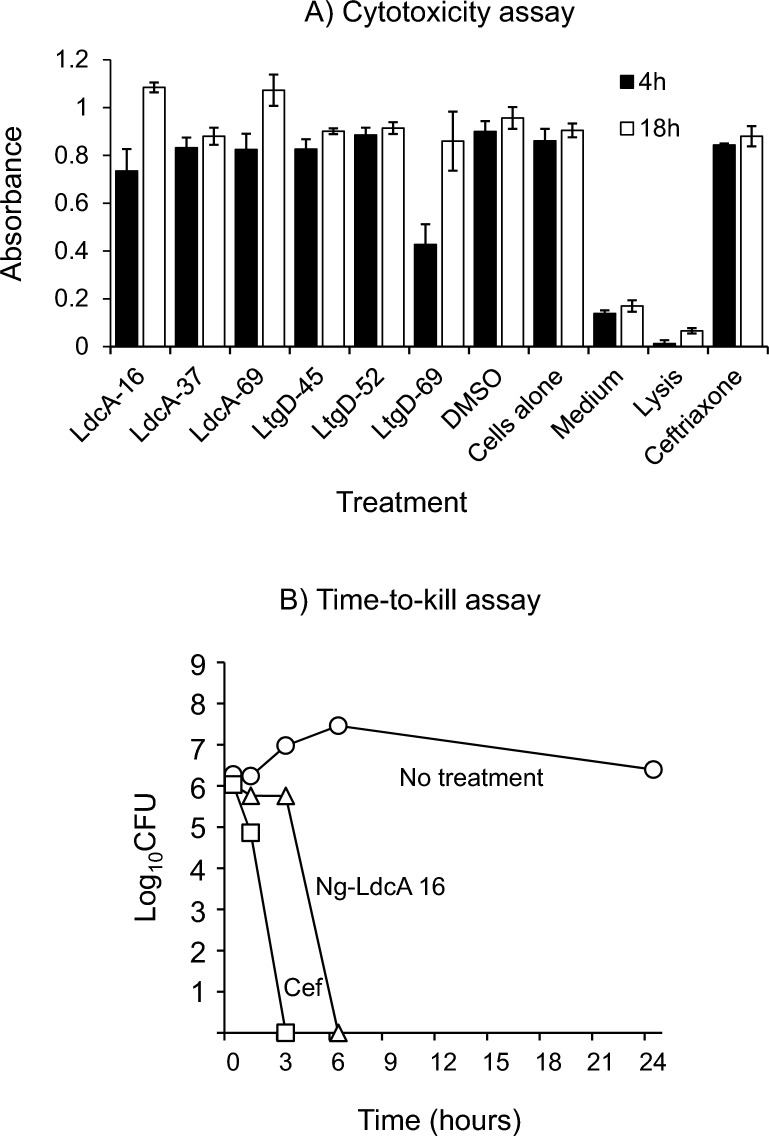
**A** Cytotoxicity of compounds. Human Chang conjunctival cells were treated with 50 µM (final concentration) of Ng-LcdA-16, -37 and -69 and Ng-LtgD-45, -52 and -69 compounds and cytotoxicity was measured using a standard resazurin assay. Controls included untreated cells, DMSO alone, medium alone, cell lysis (i.e. induced death) and ceftriaxone. The columns represent the means, and the error bars the standard error of the means of three independent experiments. **B** Determination of time to kill for compound Ng-LdcA-16. Bacteria (10^5^ CFU/well, n = 3) were treated with 50 µM (final concentration) of Ng-LdcA-16 and viable counts were made over time. Controls were bacteria alone, and bacteria treated with ceftriaxone (50 µM final concentration). Data are from one representative experiment of experiments done at least twice

We tested the cytotoxicity of the three Ng-LdcA compounds and three Ng-LtgD compounds on human Chang conjunctival epithelial cells using a standard resazurin assay, with cells treated for 18 h with 50 μM of the compounds and measurements of cell death after the addition of resazurin recorded at 4 and 18 h (Fig. 10A). There was no significant cytotoxicity shown by compounds Ng-LdcA-16, -37 and -69, nor by Ng-LtgD-45 and -52 after 4 h of cell viability measurement (P > 0.05), whereas there was a 50% reduction in cell viability recorded with Ng-LtgD-69 (P < 0.05). However, cell viability appeared to be restored when the cells were examined after 18 h, most likely due to rapid division of surviving cells unaffected by the presence of any compound.

### Specificity of the compounds for killing *N. gonorrhoeae*

We tested the hypothesis that the three Ng-LdcA compounds and three Ng-LtgD compounds did not kill bacteria of other genera and thus would be specific for gonococci. The majority of the Ng-LdcA or Ng-LtgD compounds tested at 50 µM did not kill *P. aeruginosa* or a variety of staphylococcal strains (Supplementary Table 3). Ng-LtgD-69 recorded a MIC50 of 50 µM against *S. aureus* NCTC8325.4, but when examined on other staphylococcal species, none of the Ng-LtgD compounds were active (Supplementary Table 3). Against the commensal *L. gasseri,* Ng-LdcA-69 reported a MBC50 of 50 µM and Ng-LtgD-45 and Ng-LtgD-69 compounds recorded MBC50/90 values of 12.5 µM (Supplementary Table 3). The MIC and MBC titration curves for compounds tested against *P. aeruginosa, Staphylococcus spp* and *L. gasseri* are shown in Supplementary Figs. 6, 7 and 8.

### Anti-gonococcal activity of compounds Ng-LdcA-16 and Ng-LtgD-45

Based on the initial MIC and MBC screening study and the computational modelling, we chose the best performing compounds Ng-LdcA-16 and Ng-LtgD-45 as exemplars for further biological studies. All the studies with Ng-LdcA-16 were done with compound provided by Atomwise (sourced from Enamine), whereas additional lots were necessary to complete the experiments with Ng-LtgD-45 (sourced from Chemspace and Enamine).

The ability of Ng-LdcA-16 to kill several gonococci from the FDA/CDC AR gonococcal biobank, which were reported to show increased resistance to ceftriaxone, was examined (Table 3; Supplementary Fig. 9). The reported MIC90 value for ceftriaxone for all these isolates was 0.19 µM, whereas the MIC90/MBC90 values for compound Ng-LdcA-16 were ~ 4–16 fold higher at between 0.78 and 3.125 µM. Interestingly, strain P9-17 was particularly sensitive to ceftriaxone, whereas the effects of Ng-LdcA-16 on this strain were like those observed on the FDA/CDC isolates. In time-to-kill assays, Ng-LdcA-16 killed 100% of gonococci by 6 h (Fig. 10B). For comparison, the antibiotic ceftriaxone was able to kill 100% of gonococci by 3 h (Fig. 10B).

**Table 3:** Activity of Ng-LdcA-16 against different gonococcal strains with reported resistance to ceftriaxone

Isolate bank number	Ceftriaxone MIC_90_ µM (mg/L)	Ng-LdcA-16			
		MIC50	MIC > 90	MBC50	MBC > 90
AR-0194	0.19 (0.125)	0.78 (0.26)	0.78 (0.26)	0.78 (0.26)	0.78 (0.26)
AR-0166	0.19 (0.125)	1.56 (0.5)	3.125 (1.0)	1.56 (0.5)	1.56 (0.5)
AR-0174	0.19 (0.125)	1.56 (0.5)	1.56 (0.5)	1.56 (0.5)	1.56 (0.5)
AR-0190	0.19 (0.125)	1.56 (0.5)	1.56 (0.5)	1.56 (0.5)	1.56 (0.5)
AR-0173	0.19 (0.125)	0.78 (0.26)	1.56 (0.5)	0.78 (0.26)	1.56 (0.26)
P9-17	0.003–0.006 (0.002–0.004)	< 1.56 (< 0.5)	3.123–6.25 (1.0–2.0)	0.195–0.39 (0.06–0.13)	0.39 (0.13)

MIC50—first dilution that gives ≥ 50% killing

MIC > 90—first dilution that gives > 90% killing

Reduced susceptibility to ceftriaxone is defined as MIC > 0.03 mg/L (Public Health England) and resistance to ceftriaxone is defined as MIC > 0.125mg/L (EUCAST)

In our study, the additional new lots of Ng-LtgD-45 from Chemspace and Enamine purchased to finish the remaining experiments (i.e. testing against other gonococcal strains, against other *Neisseria* spp. and time-to-kill assays), surprisingly, were not effective in the MIC and MBC assays against P9-17 gonococci growing in either supplemented GC broth or in a simple 1% (w/v) proteose peptone medium. This was not the case with any additional lots of Ng-LdcA-16. Thus, to test new batches of Ng-LtgD-45 against P9-17 and the gonococcal isolates from the FDA/CDC AR biobank, we used a bactericidal MBC assay previously used to test other compounds that were inactivated by complex growth media [53, 54]. In this established assay, we treated 10^5^ CFU of gonococci with various doses of the compound for 1 h in a PBSB solution and then examined survivors with viable counting. The MBC50 and MBC90 values are shown in Table 4 and the titration curves in Supplementary Fig. 10. In this assay, Ng-LtD-45 was highly active against all the strains, with MBC50 values ranging from 0.02–0.39 μM and MBC > 90 values from 0.05–0.78 μM (Table 4). Again, P9-17 was highly sensitive to this compound with a MBC value of 0.006 μM and MBC > 90 value of 0.048 μM. Under the test conditions, the bactericidal effects occurred within the 1 h of the exposure of the bacteria to this compound.

**Table 4:** Activity of Ng-LdcA-45 against different gonococcal strains with reported resistance to ceftriaxone

Isolate bank number	Ng-LdcA-45	
	MBC50 μM (mg/L)	MBC > 90 μM (mg/L)
AR-0194	0.02 (0.008)	0.05 (0.02)
AR-0166	0.1 (0.04)	0.39 (0.15)
AR-0174	0.1 (0.04)	0.39 (0.15)
AR-0190	0.2 (0.08)	0.39 (0.15)
AR-0173	0.39 (0.15)	0.78 (0.3)
P9-17	0.006 (0.002)	0.048 (0.02)

MBC50, MBC > 90 defined as first dilutions that give ≥ 50% and 90% killing respectively

Titration curves are shown in Supplementary Fig. 9

**Fig. 11:**
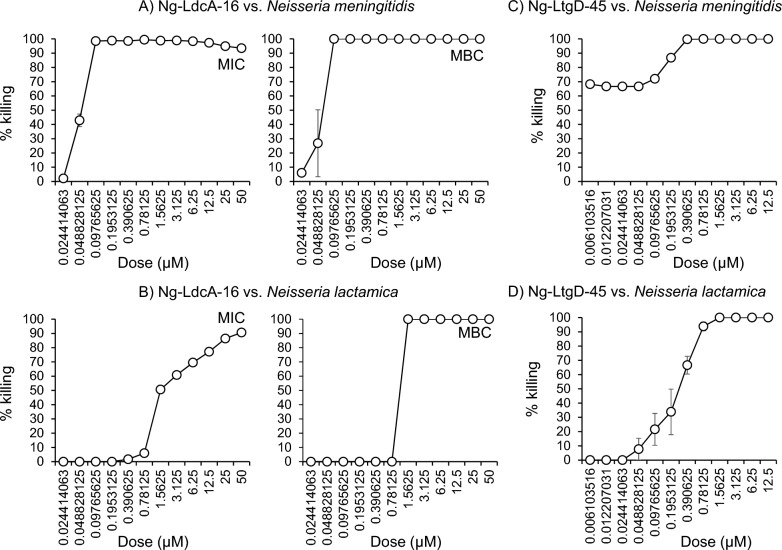
Activity of Ng-LdcA-16 and Ng-LtgD-45 compounds against other *Neisseria* spp. **A** Meningococci and **B** *N. lactamica* (10^5^ CFU/well, n = 3) were treated with various concentrations of compound Ng-LdcA-16 in the standard MIC and MBC assay, over 24 h. **C** Meningococci and **D** *N. lactamica* (10^5^ CFU/well, n = 3) were treated with various concentrations of compound Ng-LtgD-45 in the MBC assay in PBSB for 1 h with viable counting. Symbols represent the mean and any error bars the standard error of the means from three independent experiments

Finally, it is possible that the Ng-LdcA-16 and Ng-LtgD-45 compounds could have activity against other *Neisseria* spp and so they were tested against reference strains *N. meningitidis* MC58 and *N. lactamica* strain Y92-1009. Prior to this, we examined the amino acid similarities of both enzymes between the three species by Clustal. There was ~ 89% similarity in the amino acid sequences of LdcA of the three species and 94% similarity for LtgD (Supplementary Fig. 11). In addition, the amino acids belonging to the binding sites interacting with the compounds were completely conserved between the species for both LdcA and LtgD (Supplementary Fig. 11, shown in green). Thus, our hypothesis was that the two compounds would be equally effective against *N. lactamica* and *N. meningitidis.* Ng-LdcA-16 showed highly significant efficacy against *N. meningitidis* MC58 with MIC 50/ > 90 values of 0.1 μM and against *N. lactamica* with MIC50 values of 1.56 µM and MIC > 90 values of ~ 25 µM, and MBC50/90 values of 0.78—1.56 µM (Fig. 11A, B). Using the viable count assay with Ng-LtgD-45, high activity was observed against meningococci, with a MBC > 90 value of 0.39 µM, and MBC50 values < 0.01 µM (Fig. 11C). Against *N. lactamica,* MBC50 and MBC > 90 values of 0.39 µM and 0.78 µM were recorded (Fig. 11D).

## Discussion

In the current study, the AtomNet technology identified compounds within the 8 million compounds in the MCULE v20191203 library that could potentially interact with the target enzymes Ng-LdcA and Ng-LtgD. We screened the compounds in MIC and MBC assays with a gonococcal strain P9-17 and selected the top three most active compounds directed against each of the enzymes. We showed that the compounds (1) were specific for gonococci; (2) killed gonococcal isolates with reported resistance to ceftriaxone and azithromycin; (3) were bactericidal in function, with killing effects evident within 1 h of treatment; and (4) were non-toxic in a simple in vitro mammalian cell culture toxicity model. AtomNet is the world's first deep convolutional neural network for structure-based drug discovery and the literature is starting to reflect its use for hit identification [25, 62–67]. The structures of both gonococcal enzymes were not available, so AtomNet generated homology models using *E. coli* homologues. Once the compounds were tested experimentally and the most favourable hits identified, we did an in-depth computational modelling study to examine the interactions of Ng-LdcA-16, -37 and -69 and Ng-LtgD-45, -52 and -69 with their target enzymes. Our approach was underpinned by fully automated servers using artificial intelligence and deep learning algorithms to generate and analyze the large amount of generated computational data. We felt that this was important to validate the AtomNet model for the structure-based homology modelling of the enzymes and to support our biological data.

Muhammed and Aki-Yalcin stressed the importance of homology modelling in predicting protein 3D structures accurately, particularly in drug discovery [68]. They noted that the quality of the generated protein structures was crucial. Computational modelling was used to study interactions of compounds with specific enzymes, validating the accuracy of the modelling. The consistent success of this approach in different studies, prompted us to use the same for our work on proteins lacking crystal structures, underscoring its reliability in protein structure prediction as well as modelling studies. Recently Kant et al. used a similar computational modelling approach to generate a homology model for the *N. gonorrhoeae* glutamate racemase MurI protein, which also did not have a crystal structure [46]. Similarly, Pawar et al. screened natural products in silico that targeted the *Mycobacterium tuberculosis* MurI and identified the enzyme’s binding pocket and essential residues and their interactions with anti-tubercular compounds [69]. These studies serve to endorse the modelling technique used with Ng-LdcA and Ng-LtgD. Thus, we generated and validated homology models of these two enzymes, analysed and validated the active sites [70], and did molecular docking studies and analysed the dynamics of the compound interactions with the enzymes. Liao et al. have emphasized the benefits of computer-aided drug target identification methods and the significance of identifying the binding sites and evaluating ‘druggability’ [71].

Bioinformatic algorithms are used to predict binding affinities, providing initial insights into potential interactions between ligands and target proteins. These predictions require subsequent experimental validation to evaluate their accuracy. Similarly, molecular dynamics simulations predict the dynamic behaviour and potential stability of protein–ligand complexes under simulated conditions. While these predictions offer valuable information about the systems’ behaviour(s), experimental validation is essential to confirm these computational findings.

For Ng-LtgD, the AtomNet technology could identify active binding sites defined by chain A residues, and our in-depth computational analysis of active site residues and molecular docking showed that there was unanimity for the identified amino acid residues for this enzyme (Supplementary Fig. 12). A similar approach was reported by Amaral et al. to locate the active binding sites and the pivotal amino acids associated with them in the *Trypanosoma cruzi* trypanothione reductase enzyme (Tc-TR). This study demonstrated in silico the anti-parasitic potential of a natural plant product gibbilimbol B and some of its synthetic analogues against Tc-TR to support the biological findings [72]. However, the complexity of Ng-LdcA presented a distinct challenge. Our computational modelling revealed a more intricate landscape, shedding light on the nature and number of active site amino acid residues spanning both the A and B chains of the enzyme (Supplementary Fig. 12). This additional insight holds great promise for future drug modification i.e. drug repurposing studies, as it offers a deeper understanding of compound affinity and avidity interactions, potentially unlocking new avenues for drug discovery and development.

The significance of this study extends beyond computational modelling and into practical application. Both Ng-LdcA and Ng-LtgD—as integral enzymes responsible for the conversion of cell wall peptidoglycan – appear to be promising targets for the development of novel antimicrobial compounds. Our work reinforces this promise by showcasing the efficacy of lead compounds identified through virtual screening of a commercially available library using the AtomNet model. Furthermore, our study incorporated molecular dynamics, where we rigorously confirmed the stability of these novel compounds through 50 ns molecular dynamics simulations (MDS), juxtaposing them with a control molecule. The structural dynamics of the ensuing protein–ligand complexes were methodically elucidated via MM-PBSA calculations, which increased our understanding of their behaviour. The compounds exhibited not only excellent docking energy scores but also maximum stability and binding energy during simulation studies. Our approach has some similarities with the computational studies of Chaitra et al. with MmpL3, an essential membrane protein in *M. tuberculosis* [73] and from Wichapong, et al. on West Nile virus and Dengue virus NS2B/NS3 protease [74], who both used MDS to confirm the stability of protein–ligand docked complexes. In addition, Mehta et al. studied *N. meningitidis* serogroup A and its Type IV pilus assembly protein, PilF, to design an inhibitor for preventing pilus-mediated adhesion to human brain endothelial cells [75]. Our molecular docking studies focused on Ng-LdcA and Ng-LtgD served to help identify promising ligands, with strong binding affinities and stability, making them potential candidates for drug development against similar pathogens.

In our study, Ng-LdcA-16 and Ng-LtgD-45 were the most active compounds of each triplicate set against gonococci. To our knowledge, the only compound to be tested previously against Ng-LdcA was a dithiazoline compound JNJ-853346 (DTZ), which has been reported to be bacteriostatic for gonococci, but not by inhibiting Ng-LdcA enzyme activity [76]. DTZ (*Mr* = 275) had reported MIC values ranging from 116 to 233 μM (= 32–64 mg/L) against the WHO panel of reference strains. In our study, we did not test our compounds against any of the WHO panel strains, but instead against isolates from the CDC/FDA gonococcal bank with reported resistance to ceftriaxone and azithromycin. Thus, although a direct comparison was not possible of the bactericidal efficacy of our compounds with DTZ, as the isolates tested were different, compound Ng-LdcA-16 had MIC50/90 values in the range of 0.78–3.125 μM against the CDC/FDA isolates. Moreover, this range was marginally inferior to the reported activity of ceftriaxone (0.19 μM against these strains) and these data suggest that Ng-LdcA-16 is a potentially good candidate for further pharmacological and toxicological studies. By contrast, we could find no literature on compounds directed against Ng-LtgD that have been tested for anti-gonococcal bactericidal/bacteriostatic activity. Our study did show that both Ng-LdcA and Ng-LtgD are present in other *Neisseria* species, and relatively well-conserved. Accordingly, both Ng-LdcA-16 and Ng-LtgD-45 (using the modified bactericidal assay) were bactericidal towards *N. meningitidis* and *N. lactamica*.

The thin peptidoglycan layer is essential for maintaining cell shape, structure integrity and internal osmotic pressure of the gonococcus [57], and ~ 50% of peptidoglycan is recycled during each round of cell division [77, 78]. The Ng-LtgD enzyme maintains homeostatic levels of peptidoglycan incorporation into the layer [20] and produces the majority of released fragments [19], and deletion of the *ltgd* gene has been shown to reduce the release of peptidoglycan monomers [19]. The Ng-LdcA enzyme converts cell wall tetrapeptide-stem peptidoglycan to release tripeptide-stem peptidoglycan and is thus involved also in the recycling process [18]. In *E. coli*, deletion of the similar LdcA enzyme resulted in a buildup of uridine diphosphate-N-acetylmuramic acid-tetrapeptide and autolysis of the bacteria when they reached stationary phase [79, 80]. Furthermore, in the gonococcus, mutations that disabled the active site of Ng-LdcA disrupted cellular homeostasis and recycling, by altering the ratio of released peptidoglycan fragments from 3:1 tripeptide:tetrapeptide monomers to almost only tetrapeptide monomers [18]. Despite these perturbations, deletion of *ltgd* and *ldca* do not affect bacterial viability, and gonococcal mutants were able to grow on solid media and in liquid media. Examining the bactericidal mechanism(s) of Ng-LdcA-16 and Ng-LtgD-45 was outside the scope of the current study, though it is possible to postulate how these compounds may exert their biological effects on gonococci. Our in silico structural and molecular dynamics predictions demonstrated that the compounds could bind to their target enzymes, which was accompanied by highly significant reductions in gonococcal numbers following exposure to the compounds in vitro. Thus, a potential mechanism is that binding of Ng-LdcA and/or Ng-LtgD with target enzymes accumulates negative impacts on cell wall homeostasis and peptidoglycan recycling that leads to cell lysis; these effects could possibly occur over a 24 h period in the standard MIC and MBC assays. However, the fact that deletion mutants are still viable, and that compound Ng-LtgD-45 can kill gonococci within 1 h of treatment suggests alternative mechanism(s), e.g. a potentially lytic effect on the cell membrane. Testing these hypotheses would require future studies that examine membrane damage effects alongside perturbations to peptidoglycan recycling and fragment production following treatment.

Our computational studies did not give any indication on solvent stability of the compounds, and the importance of doing wet laboratory work was demonstrated by the observation that Ng-LtgD-45 showed significant batch-to-batch variability in efficacy, which was not observed with Ng-LdcA. It was evident that bacterial growth media could inactivate batches of Ng-LtgD-45, and the bactericidal effect could only be observed with the different batches using a modified bactericidal assay. Chemical instability of Ng-LtgD-45 must be overcome to develop this compound as a treatment, and this could be done by bringing together the computational and laboratory approaches to design chemically altered analogues in silico, model their interactions in silico, followed by direct testing against the pathogen.

## Conclusions

From (1) initial identification with a deep convolutional neural network for structure-based drug discovery; (2) validation with comprehensive computational modelling studies, residue interaction analyses, and dynamic simulations, followed by; (3) wet laboratory testing against bacteria, we have identified biocidal compounds targeting the *N. gonorrhoeae* LdcA and LtgD enzymes. Taking all data into consideration, we propose that compound Ng-LdcA-16 is a promising anti-gonococcal compound for further development, and that Ng-LtgD-45 requires further analogue development and testing. Further work to overcome limitations to our study could include structural co-crystallization studies of compounds binding with their target enzymes, additional toxicological and resistance studies, e.g. using the hollow fiber model [81] and in vivo efficacy studies with the mouse gonorrhoea model.

## Supplementary Information

Additional file 1: Figure S1. Minimum inhibitory concentration (MIC) titration curves for Ng-LdcA compounds. Compound 35 could not be titrated (MIC value < 50 μM). S2. Minimum inhibitory concentration (MIC) titration curves for Ng-LtgD compounds. Compounds 68 and 74 could not be titrated (MIC values < 50 μM). S3. Titration of compounds to determine minimum bactericidal concentrations (MBC) for Ng-LdcA and Ng-LtgD compounds. Data are representative of n = 2 experiments for the best-performing compounds with most active MICs. S4. Sequence alignment of Ng-LtgD with *E. coli* 1D0K at the C-terminus, highlighting the modelled regions and indicating the excluded parts. The position of the modelled region start is also shown. S5. Ramachandran plots. To assess model accuracy and stereo-chemical properties of Ng-LdcA and Ng-LtgD. S6. MIC and MBC titration curves for Ng-LdcA and Ng-LtgD compounds tested against *P. aeruginosa* PAO-1. Bacteria (10^5^ CFU/well, n = 3) were treated with various concentrations of compounds Ng-LdcA-16, -37 and -69 and Ng-LtgD-45, -52 and -69 in the standard MIC and MBC assays. Data are representative of n = 2 experiments. S7. MIC and MBC titration curves for Ng-LdcA and Ng-LtgD compounds tested against *Staphylococcus spp.* Bacteria (10^5^ CFU/well, n = 3) were treated with various concentrations of compounds Ng-LdcA-16, -37 and -69 and Ng-LtgD-45, -52 and -69 in the standard MIC and MBC assays. Data are representative of n = 2 experiments. MBC experiments were only done with compounds that showed detectable MIC50 values. S8. MIC and MBC titration curves for Ng-LdcA and Ng-LtgD compounds tested against *Lactobacillus gasseri.* Bacteria (10^5^ CFU/well, n = 3) were treated with various concentrations of compounds Ng-LdcA-16, -37 and -69 and Ng-LtgD-45, -52 and -69 in the standard MIC and MBC assays. Data are representative of n = 2 experiments. MBC experiments were done only with 50 μM concentrations. S9. MIC and MBC titration curves for Ng-LdcA-16 tested against different gonococci belonging to the FDA/CDC AR gonococcal biobank. Bacteria (10^5^ CFU/well, n = 3) were treated with various concentrations of compounds Ng-LdcA-16, in the standard MIC and MBC assays. Data curves are representative of n = 2 experiments. S10. MBC assays for Ng-LtgD-45 against strain P9-17 and other gonococci belonging to the FDA/CDC AR gonococcal biobank. Bacteria (10^5^ CFU/well) were treated with various concentrations of compound Ng-LtgD-45 in the MBC assay in PBSB for 1h with variable counting. Symbols represent the mean and any error bars the standard error of the means from three independent experiments. S11. Clustal alignments of the Ng-LdcA and Ng-LtgD enzymes from three* Neisseria* spp. The green highlighted amino acids belong to the binding sites of the enzymes that interact with the compounds. S12. Alignment of active site amino acid residues for Ng-LdcA and Ng-LtgD, determined by AtomNet and our computational modelling. Yellow highlight denotes the shared amino acids identified by both algorithms.

Additional file 2: Table S1. MIC and MBC values for all Ng-LdcA compounds tested against *Neisseria gonorrhoeae* P9-17. Table shows all compounds tested with their pre-screen values and titration values, MCULE ID, product URL and SMILES. The rows highlighted in yellow show the three compounds chosen for further computational modelling and in vitro studies. nd = no detectable killing; MIC, minimum inhibitory concentration; MBC, minimum bactericidal concentration; 41 is control DMSO; SMILES, simplified molecular-input line-entry system; ID and product url available from Mcule.com online drug discovery platform. S2. MIC and MBC values for all Ng-LtgD compounds tested against *Neisseria gonorrhoeae* P9-17. Table shows all compounds tested with their pre-screen values and titration values, MCULE ID, product URL and SMILES. The rows highlighted in yellow show the three compounds chosen for further computational modelling and in vitro studies. nd = no detectable killing; MIC, minimum inhibitory concentration; MBC, minimum bactericidal concentration; 66 is control DMSO; SMILES, simplified molecular-input line-entry system; ID and product url available from Mcule.com online drug discovery platform. S3. Specificity of compounds: summary of MIC and MBC for compounds tested against other bacteria. The top three Ng-LdcA and Ng-LtgD compounds tested against other bacteria. Values are generated from at least n = 2 MIC and MBC experiments. See Supplementary figures for titration curves for MIC and MBC experiments.

## Data Availability

All data pertaining to this article are included herein and within the supplementary material.
