# Supplementary material for: Antimicrobial activity of compounds identified by artificial intelligence discovery engine targeting enzymes involved in Neisseria gonorrhoeae peptidoglycan metabolism
Source: Biol Res. 2024 Sep 5;57:62. doi: 10.1186/s40659-024-00543-9 (PMC11375863; doi:10.1186/s40659-024-00543-9)
Supplement: Supplementary file 1 — Additional file 1: Figure S1. Minimum inhibitory concentration (MIC) titration curves for Ng-LdcA compounds. Compound 35 could not be titrated (MIC value < 50 μM). S2. Minimum inhibitory concentration (MIC) titration curves for Ng-LtgD compounds. Compounds 68 and 74 could not be titrated (MIC values < 50 μM). S3. Titration of compounds to determine minimum bactericidal concentrations (MBC) for Ng-LdcA and Ng-LtgD compounds. Data are representative of n = 2 experiments for the best-performing compounds with most active MICs. S4. Sequence alignment of Ng-LtgD with E. coli 1D0K at the C-terminus, highlighting the modelled regions and indicating the excluded parts. The position of the modelled region start is also shown. S5. Ramachandran plots. To assess model accuracy and stereo-chemical properties of Ng-LdcA and Ng-LtgD. S6. MIC and MBC titration curves for Ng-LdcA and Ng-LtgD compounds tested against P. aeruginosa PAO-1. Bacteria (105 CFU/well, n = 3) were treated with various concentrations of compounds Ng-LdcA-16, -37 and -69 and Ng-LtgD-45, -52 and -69 in the standard MIC and MBC assays. Data are representative of n = 2 experiments. S7. MIC and MBC titration curves for Ng-LdcA and Ng-LtgD compounds tested against Staphylococcus spp. Bacteria (105 CFU/well, n = 3) were treated with various concentrations of compounds Ng-LdcA-16, -37 and -69 and Ng-LtgD-45, -52 and -69 in the standard MIC and MBC assays. Data are representative of n = 2 experiments. MBC experiments were only done with compounds that showed detectable MIC50 values. S8. MIC and MBC titration curves for Ng-LdcA and Ng-LtgD compounds tested against Lactobacillus gasseri. Bacteria (105 CFU/well, n = 3) were treated with various concentrations of compounds Ng-LdcA-16, -37 and -69 and Ng-LtgD-45, -52 and -69 in the standard MIC and MBC assays. Data are representative of n = 2 experiments. MBC experiments were done only with 50 μM concentrations. S9. MIC and MBC titration curves for Ng-LdcA-16 tested agains [file 40659_2024_543_MOESM1_ESM.pdf]

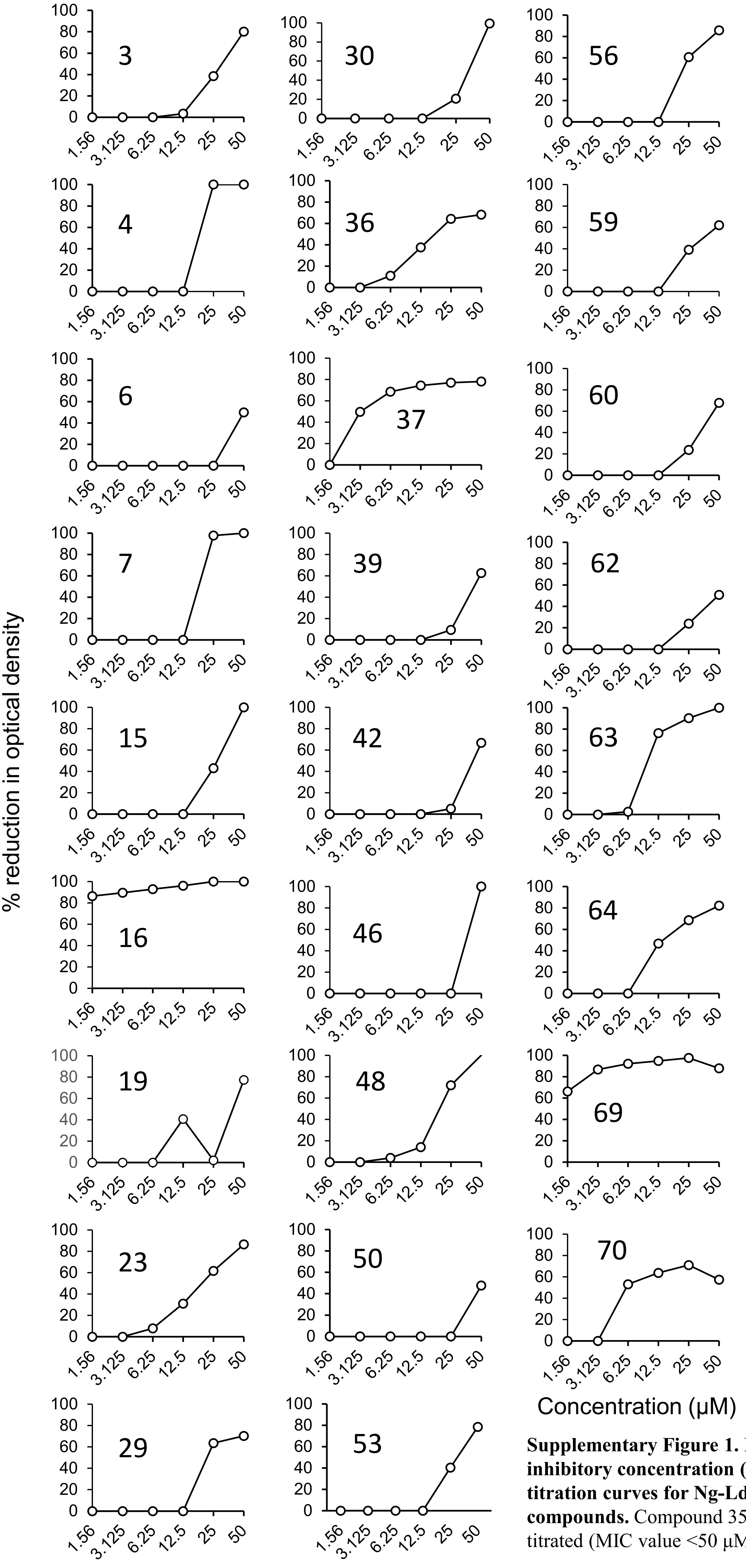

Supplementary Figure 1. Minimum inhibitory concentration (MIC) titration curves for Ng-LdcA compounds. Compound 35 could not be titrated (MIC value <50 µM).

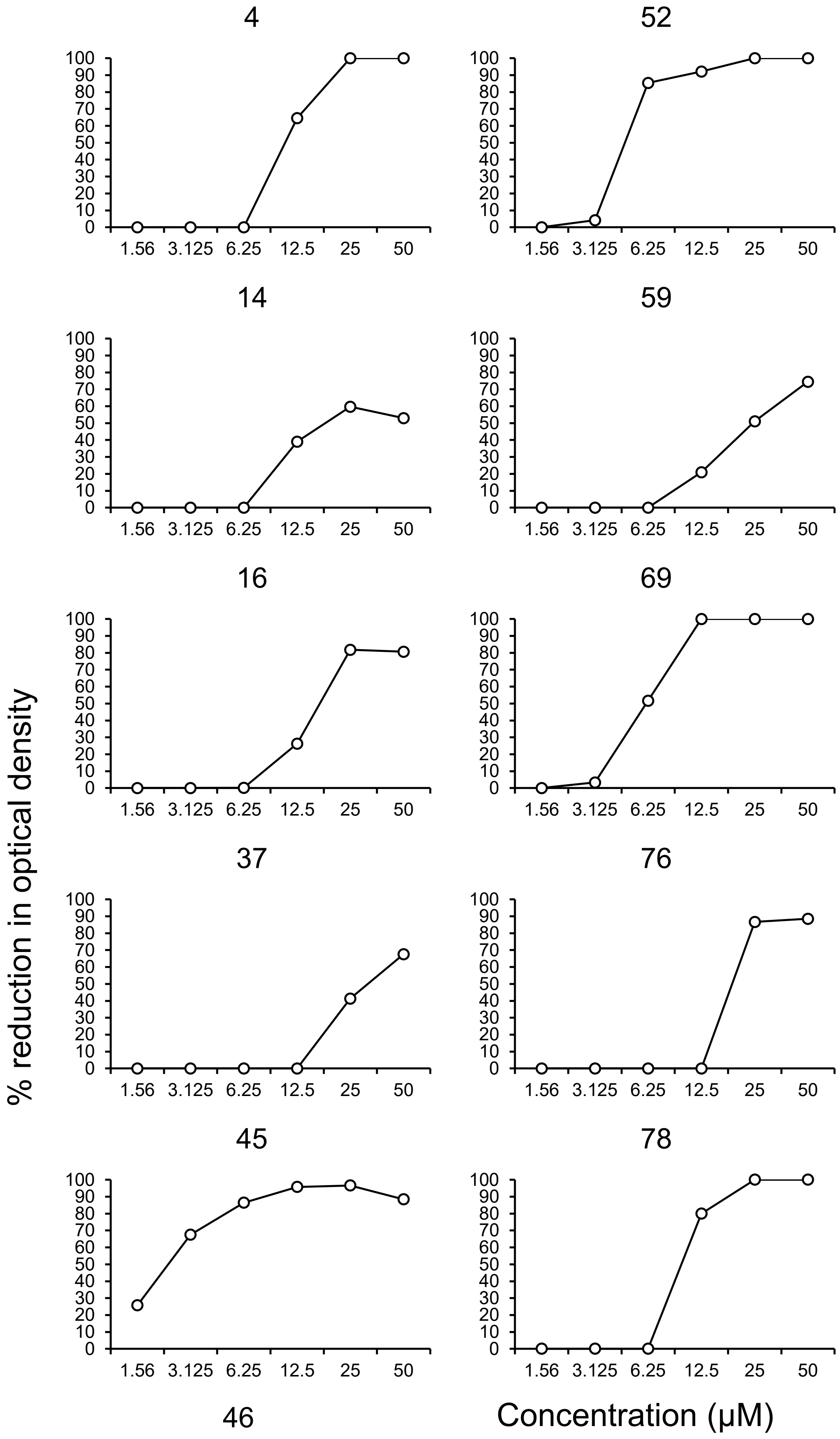

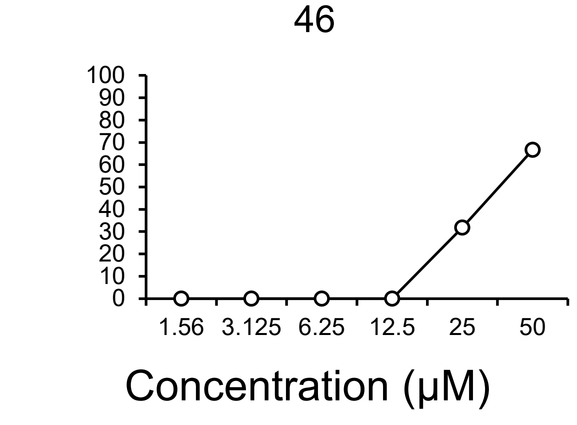

Supplementary Figure 2. Minimum inhibitory concentration (MIC) titration curves for LtgD compounds. Compounds 68 and 74 could not be titrated (MIC values < 50µM).

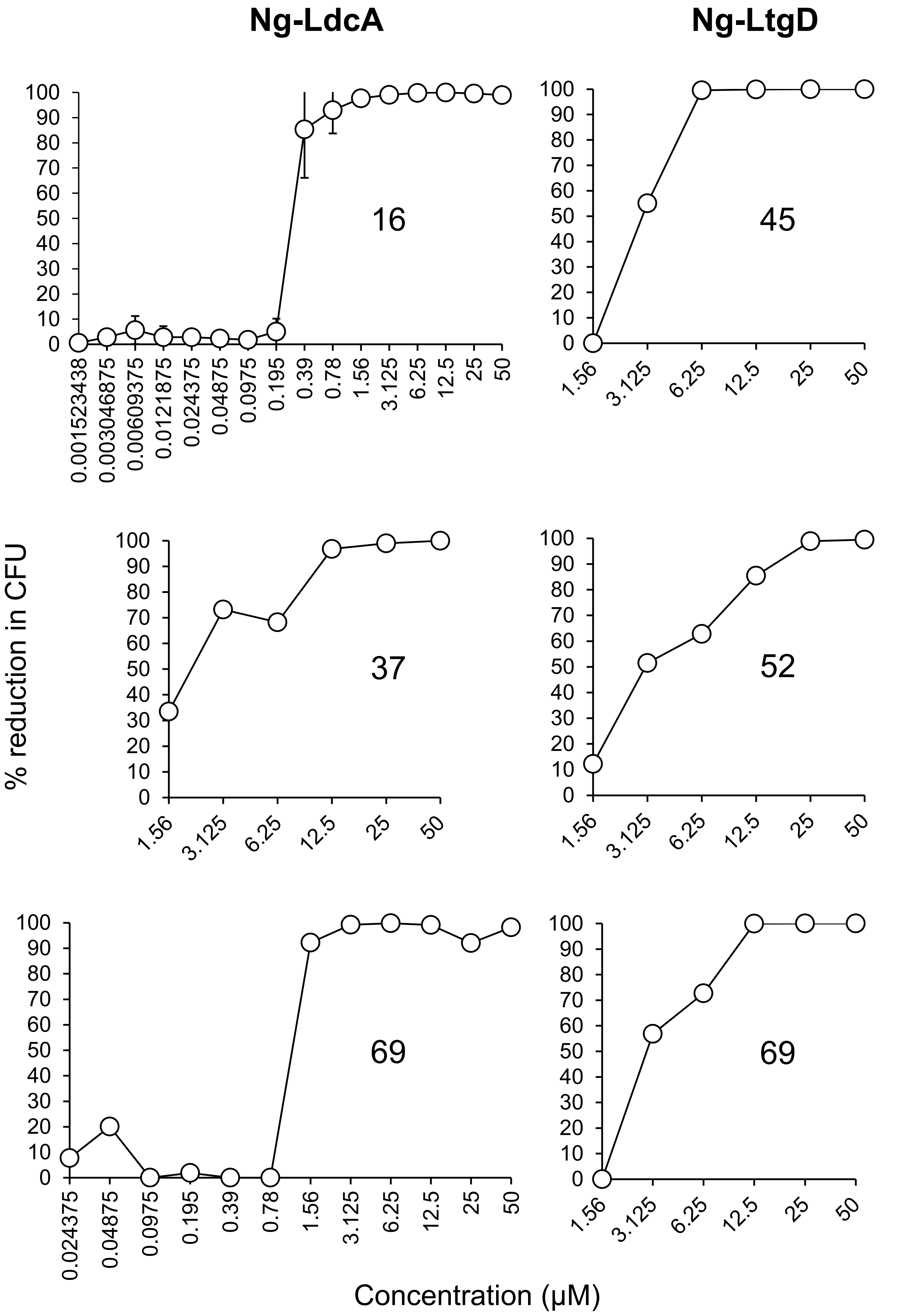

Supplementary Figure 3. Titration of compounds to determine minimum bactericidal concentrations (MBC) for Ng-LdcA and Ng-LtgD compounds . Data are representative of n=2 experiments for the best-performing compounds with most active MICs.

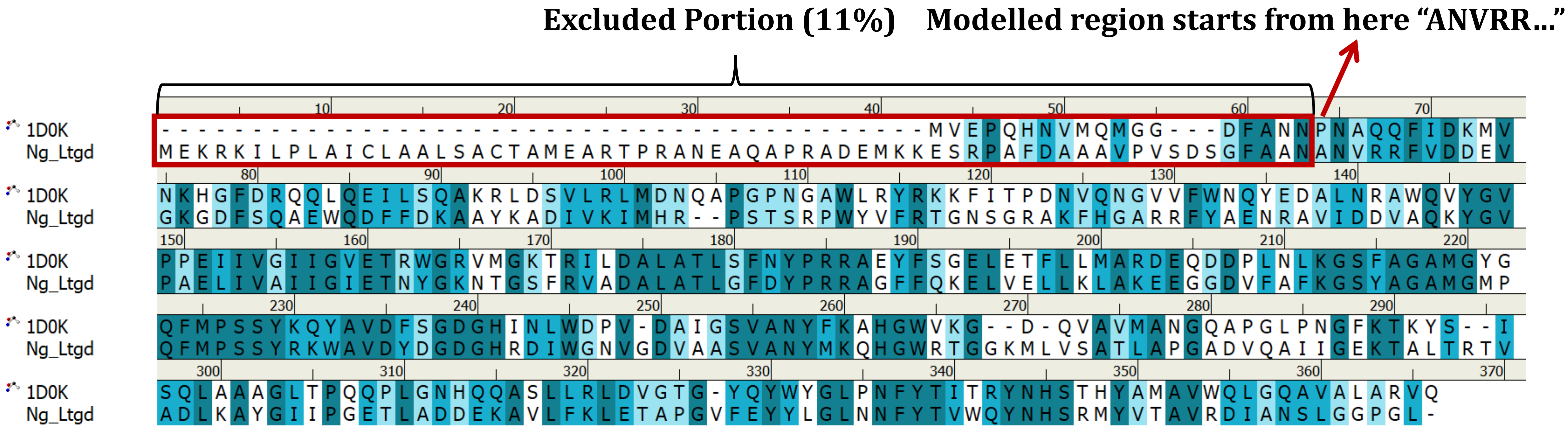

Supplementary Figure 4. Sequence alignment of Ng-LtgD with *E. coli* 1D0K at the C-terminus, highlighting the modelled regions and indicating the excluded parts. The position of the modelled region start is also shown.

Supplementary Figure 5. Ramachandran plots. To assess model accuracy and stereo-chemical properties of Ng-LdcA and Lg-LtgD.

Ng-LdcA

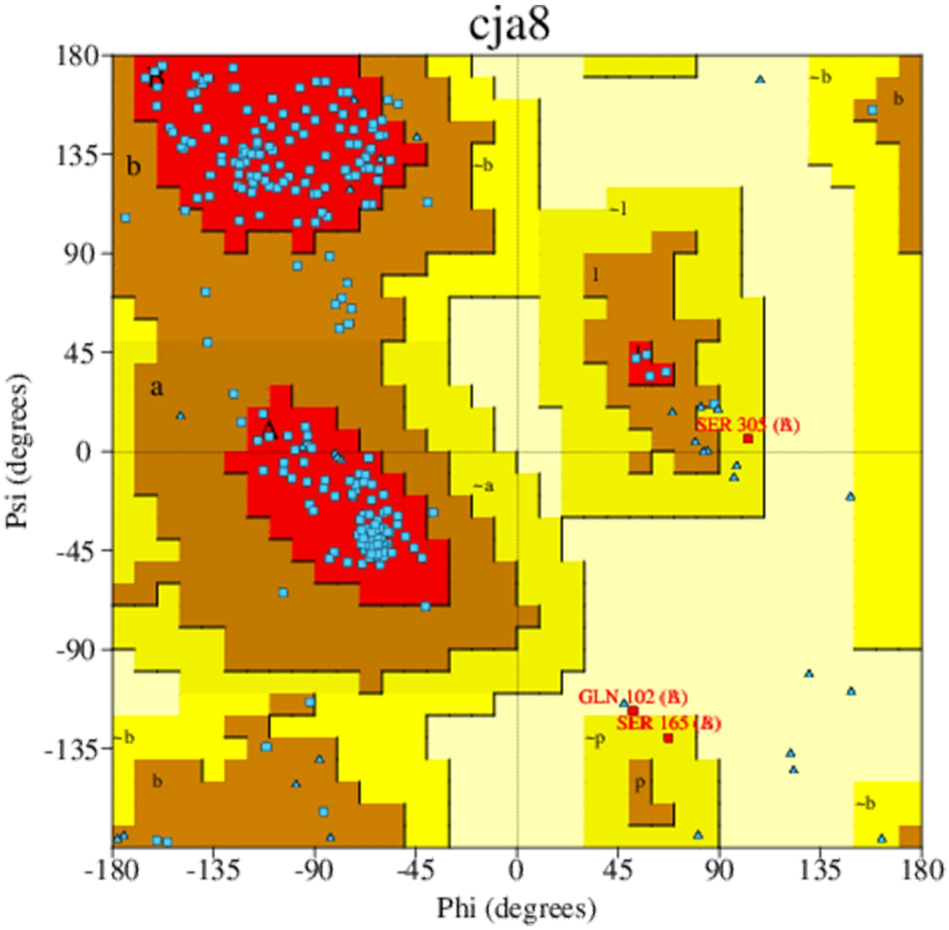

Ng-LtgD

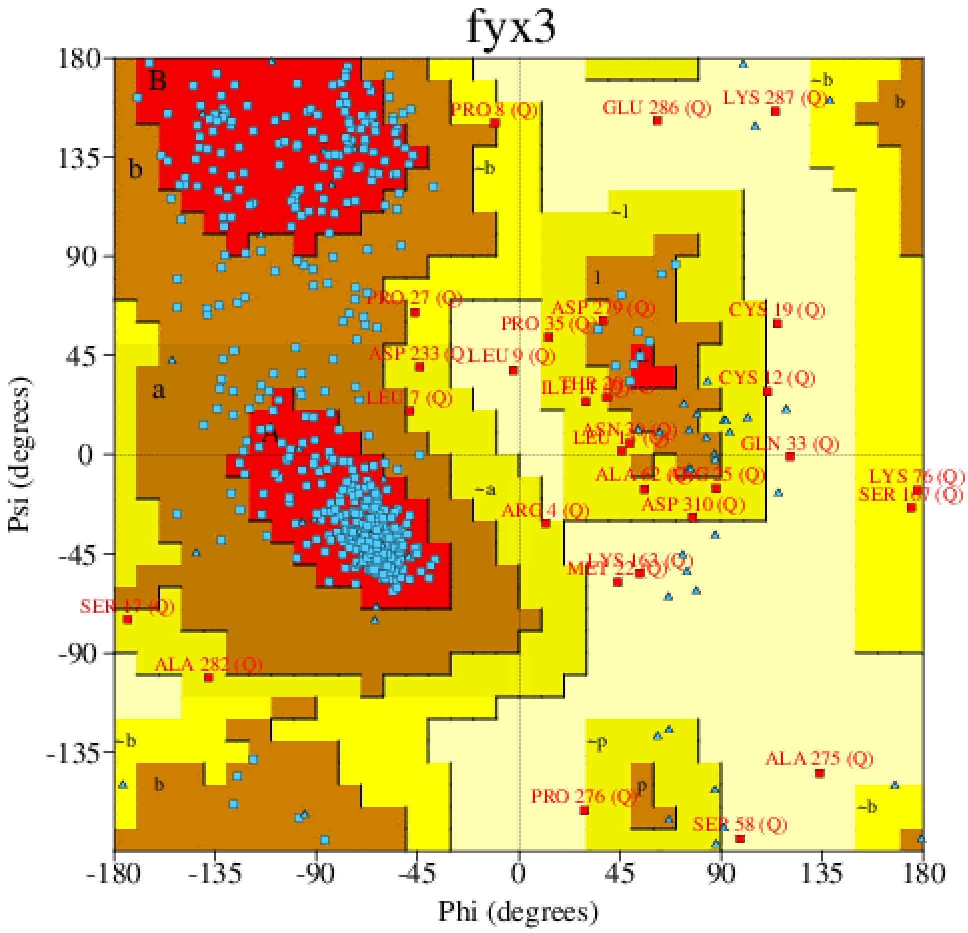

Supplementary Figure 6. MIC and MBC titration curves for Ng-LdcA and Ng-LtgD compounds tested against *P. aeruginosa* PAO-1. Bacteria (10^5 CFU/well, n=3) were treated with various concentrations of compounds Ng-LdcA-16, -37 and -69 and Ng-LtgD-45, -52 and -69 in the standard MIC and MBC assays. Data are representative of n=2 experiments.

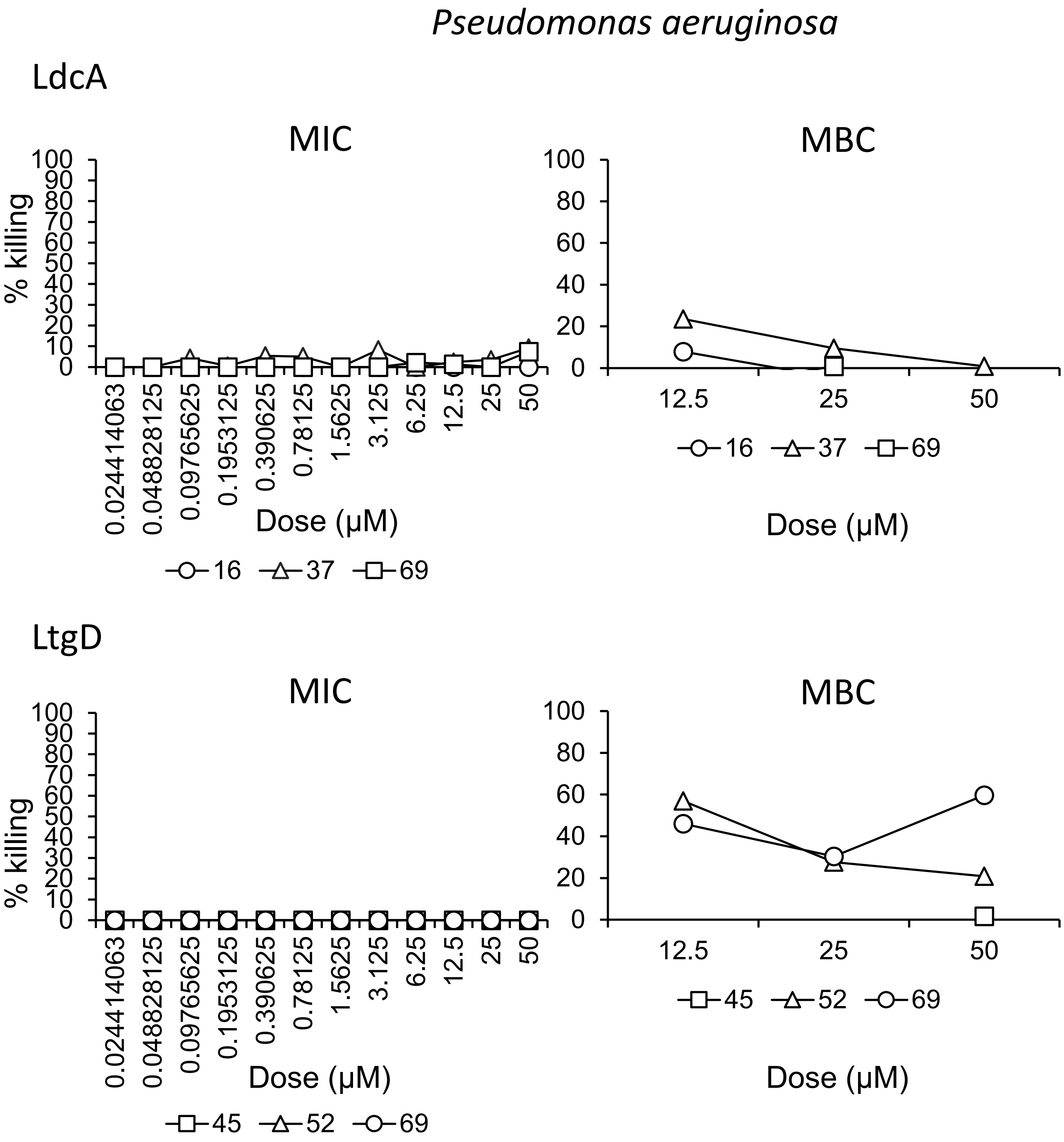

A)

Ng-LdcA *Staphylococcus aureus* NCTC8325.4

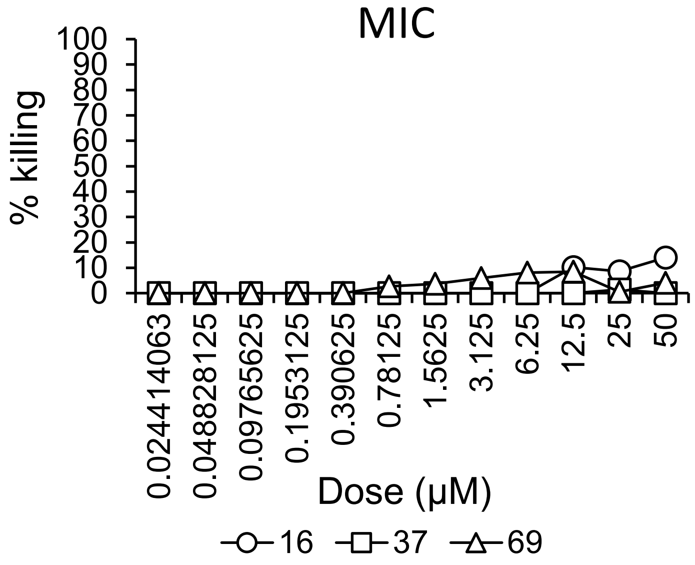

Supplementary Figure 7. MIC and MBC titration curves for Ng-LdcA and Ng-LtgD compounds tested against *Staphylococcus* spp. Bacteria (10^5 CFU/well, n=3) were treated with various concentrations of compounds Ng-LdcA-16, -37 and -69 and Ng-LtgD-45, -52 and -69 in the standard MIC and MBC assays. Data are representative of n=2 experiments. MBC experiments were only done with compounds that showed detectable MIC50 values.

Dose (µM)

Ng-LtgD

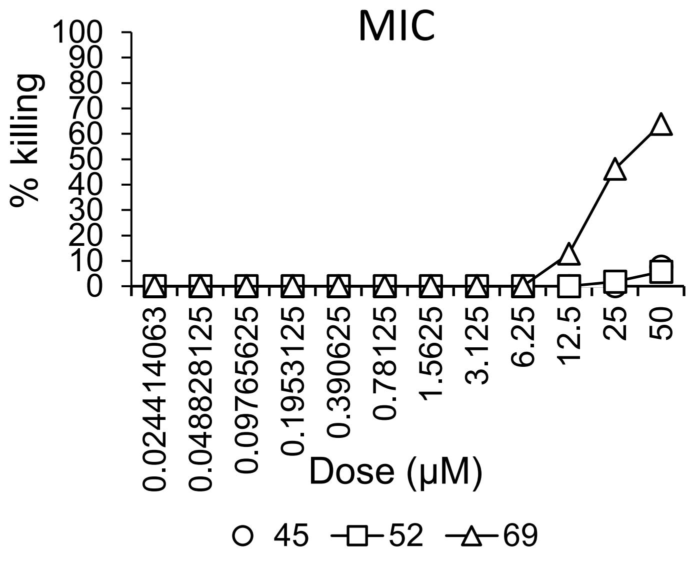

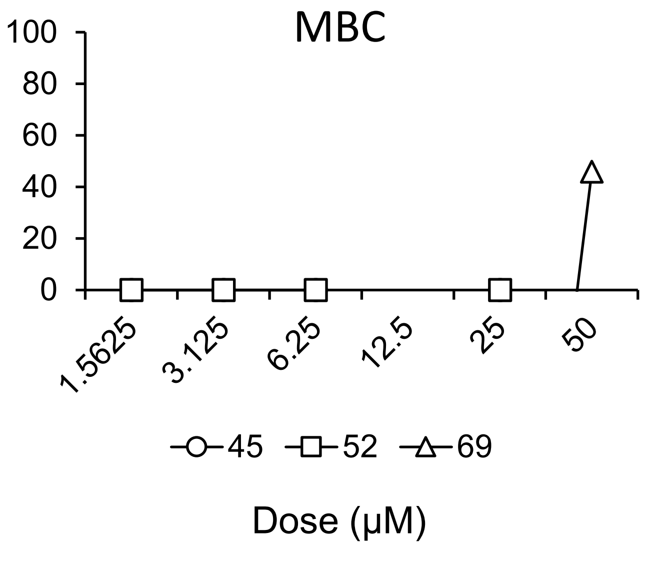

MIC of Compound Ng-LtgD-69 against other *Staphylococcus spp*.

B)

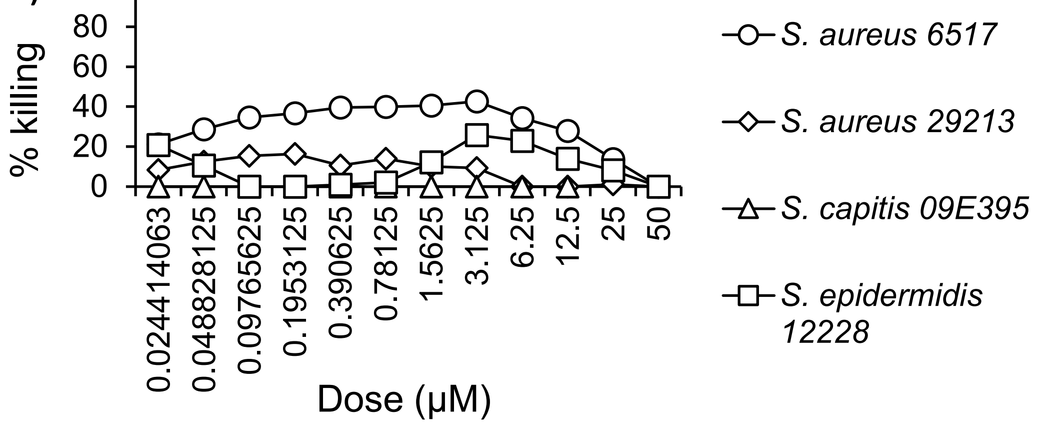

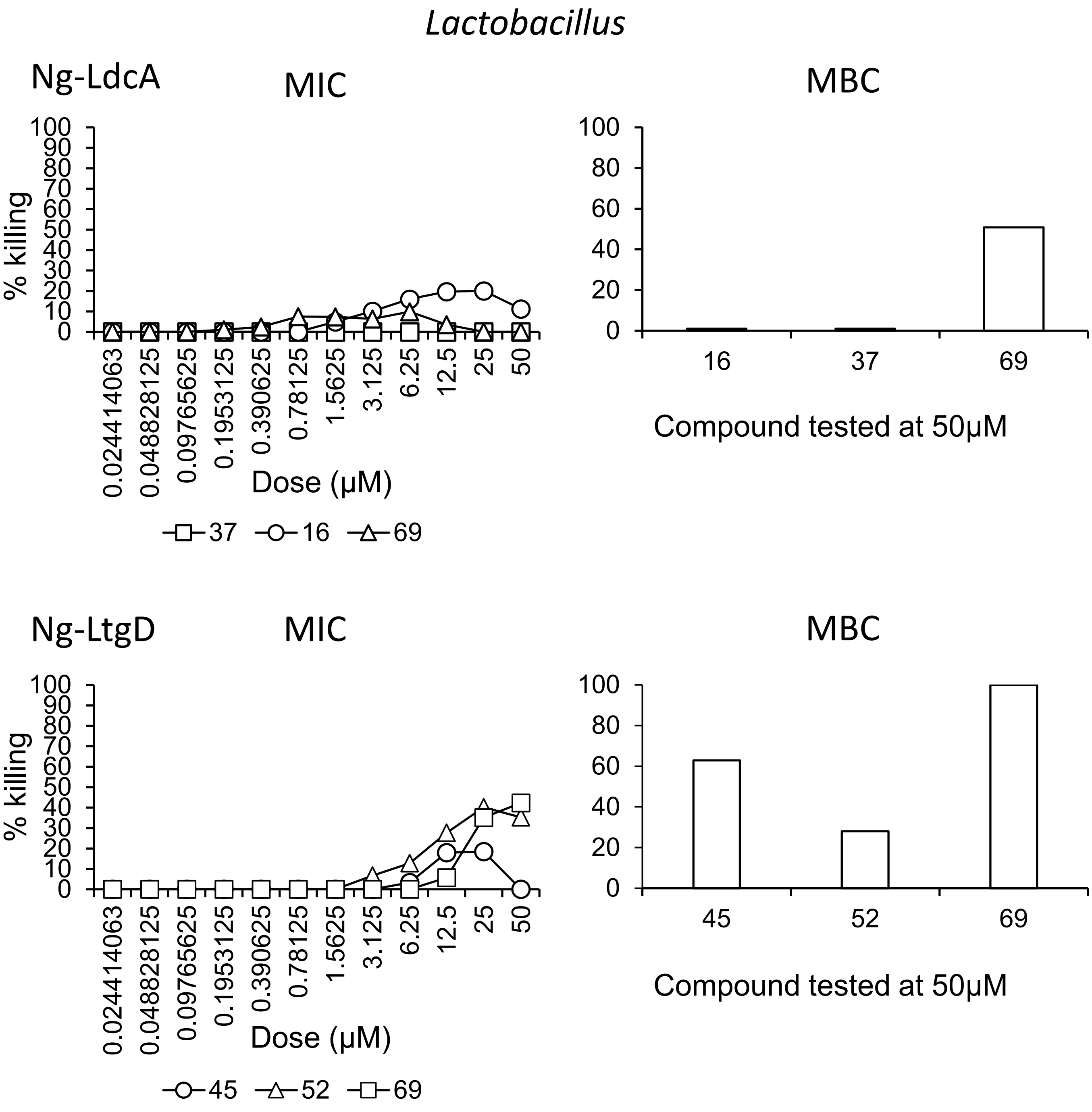

Supplementary Figure 8. MIC and MBC titration curves for Ng-LdcA and Ng-LtgD compounds tested against *Lactobacillus gasseri*. Bacteria (10^5 CFU/well, n=3) were treated with various concentrations of compounds Ng-LdcA-16, -37 and -69 and Ng-LtgD-45, -52 and -69 in the standard MIC and MBC assays. Data are representative of n=2 experiments. MBC experiments were one only with 50 µM concentrations.

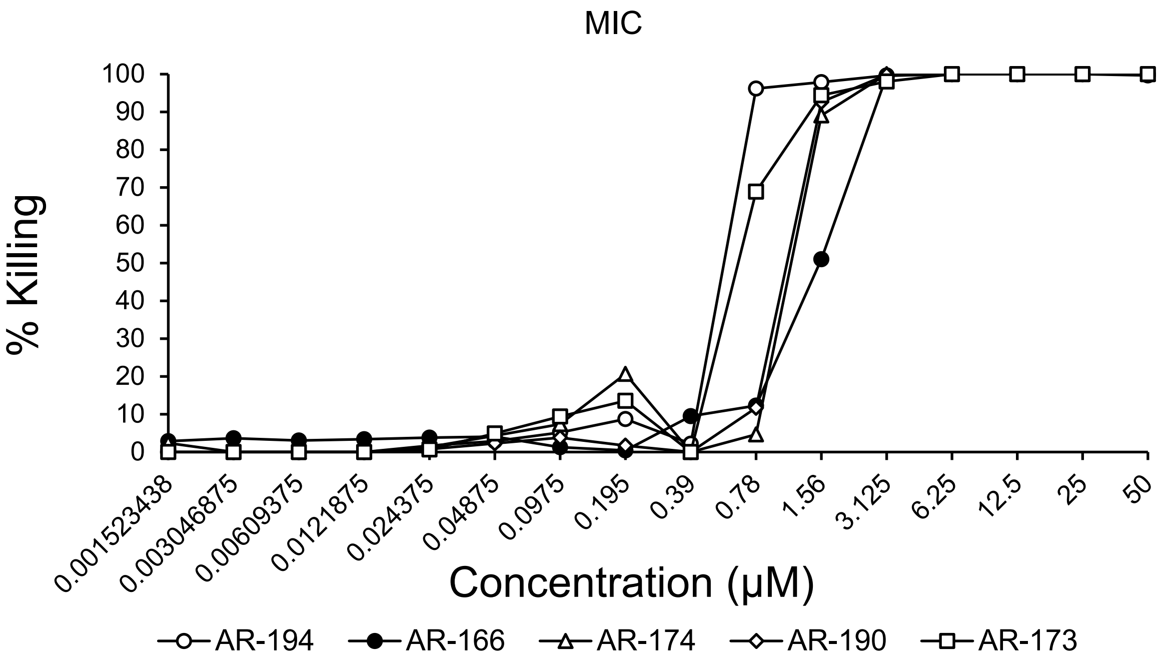

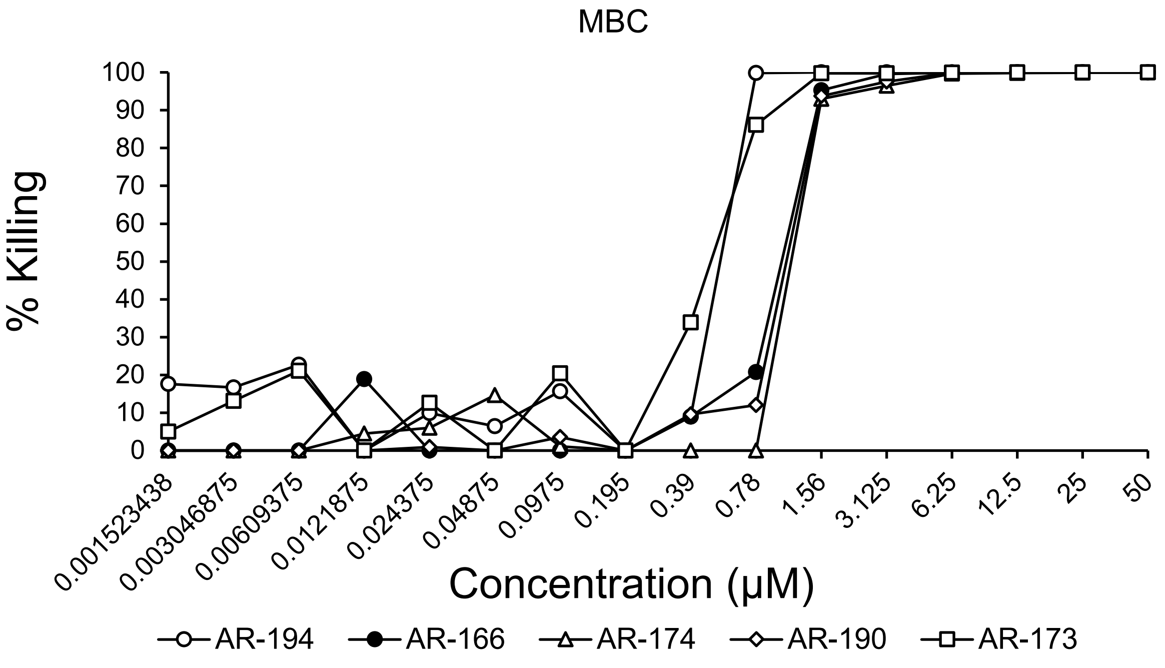

Supplementary Figure 9. MIC and MBC titration curves for Ng-LdcA-16 tested against different gonococci belonging to the FDA/CDC AR gonococcal biobank. Bacteria (10^5 CFU/well, n=3) were treated with various concentrations of compounds Ng-LdcA-16, in the standard MIC and MBC assays. Data curves are representative of n=2 experiments.

A) MBC assay for Ng-LtgD-45 vs. P9-17

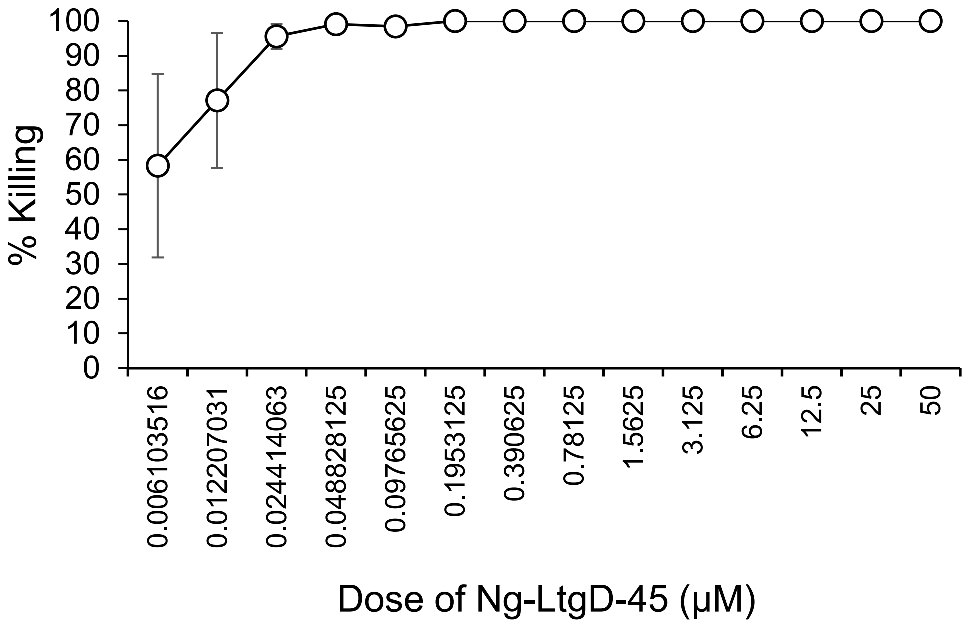

B) MBC assay for Ng-LtgD-45 vs. other gonococcal strains

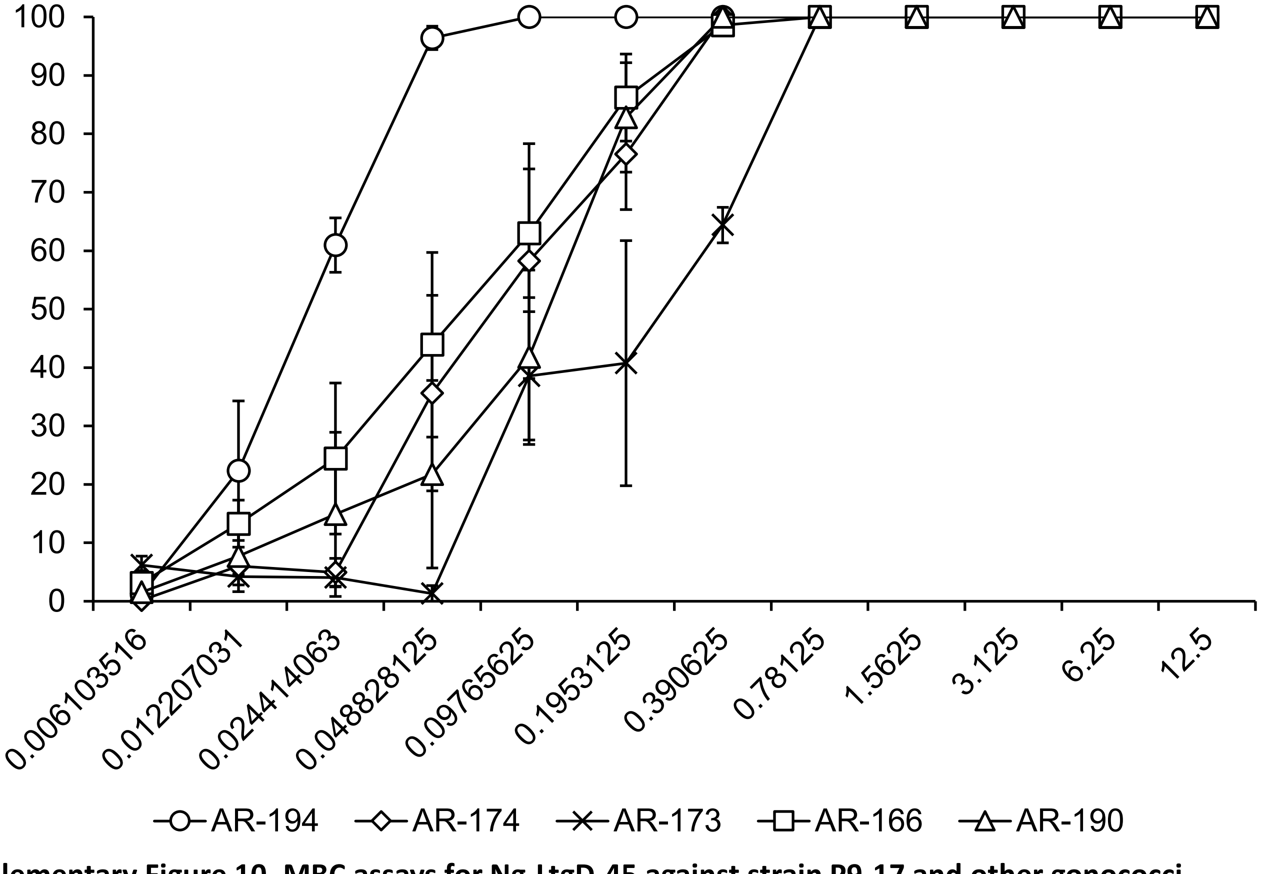

Supplementary Figure 10. MBC assays for Ng-LtgD-45 against strain P9-17 and other gonococci belonging to the FDA/CDC AR gonococcal biobank. Bacteria (10^5 CFU/well) were treated with various concentrations of compound Ng-LtgD-45 in the MBC assay in PBSB for 1h with variable counting. Symbols represent the mean and any error bars the standard error of the means from three independent experiments.

Supplementary Figure 11. Clustal alignments of the Ng-LdcA and Ng-LtgD enzymes from three *Neisseria* spp.

1. **Ng-LdcA (NEIS1546/NGO1274)**

```
4945_Nlac_Allele3      MIEPTSRRRFLKTCTAAAGAGLLQACGTAPASNNAPSAPSHPVAKARTVYPKPPRHSNSA  60
36675_Ngo_P9-17        MTEPTSRRRFLKTCTA-AGAGLLQACGTSATS-VPPLPSSHSVVKARTVTLQTPRRQSSD  58
240_Nmen_MC58          MTEPTSRRRFLKTCTAAAGAGLLQACGTSATS-VPPLPSSHSVVKARTVPLQTPRRQSSD  59
                       * ************** ***********: :*   *   ** *.*****  : **:..*

4945_Nlac_Allele3      DNLLRIVASSGFAEDTNRVNTALTRLYNAGFTVTNQQAGSRRFQRFAGTDAQRAADFQDV  120
36675_Ngo_P9-17        GNLLRVVASSGFAEDTNRVNTALTRLYNAGFTVTNQQAGSRRFQRFAGTDAQRAADFQEV  118
240_Nmen_MC58          GNLLRVVASSGFAEDTNRVNTALTRLYNVGFTVTNQQAGSRRFQRFAGTDTQRAADFQEV  119
                       .****:**********************.*********************:*******:*

4945_Nlac_Allele3      ASGRVATPKVLMGLRGGYGAARILPHIDFASLGARMRERGTLFFGFSDVCAVQLALLAKG  180
36675_Ngo_P9-17        ASGRVATPKVLMGLRGGYGAARILPHIDFASLGARMREHGTLFFGFSDVCAVQLALLAKG  178
240_Nmen_MC58          ASGRVATPKVLMGLRGGYGAARILPHIDFASLGARMREHGTLFFGFSDVCAVQLALLAKG  179
                       **************************************:*********************

4945_Nlac_Allele3      NMMSFAGPMAYSEFGKPSPSVFTMDAFIKGATQNRLTVDVPYIQRANVETEGTLWGGNLS  240
36675_Ngo_P9-17        NMMSFAGPMAYSDFGKPAPGAFTMDAFIKGATQNRLTVDVPYIQRADVETEGTLWGGNLS  238
240_Nmen_MC58          NMMSFAGPMAYSDFGKPAPGAFTMDAFIKGATQNRLTVDVPYIQRADVETEGILWGGNLS  239
                       ************:****:*..*************************:***** *******

4945_Nlac_Allele3      VLASLAGTPYMPDIDGGILFLEDVGEQPYRIERMLNTLYLSGILDKQRAIVFGDFRMGNI  300
36675_Ngo_P9-17        VLASLAGTPYMPDIDGGILFLEDVGEQPYRIERMLNTLYLSGILGKQRAIVFGDFRMEKI  298
240_Nmen_MC58          VLASLAGTPYMPDIDGGILFLEDVGEQPYRIERMLNTLYLSGILKKQRAIVFGNFRMEKI  299
                       ******************************************** ********:*** :*

4945_Nlac_Allele3      RDVYDSSYDFSTVVNHISRTAKIPVLTGFPFGHIADKITFPLGAHARIRMNGNGGYSVAF  360
36675_Ngo_P9-17        RDLYDSSYDFSAVAKHISRTAKIPVLTGFPFGHIADKITFPLGAHTRIRMNGNGGYSVAF  358
240_Nmen_MC58          RDVYDPSYDFSAVANHVSRTAKIPVLTGFPFGHIADKITFPLGAHARIRMNGNSGYSVAF  359
                       **:** *****:*.:*:****************************:*******.******

4945_Nlac_Allele3      EGYPTLDASALTLDTLLPPPNLPIFPESGVADISE  395
36675_Ngo_P9-17        EGYPTLDASALTLDTLLPPPDLPIFPESGVADISE  393
240_Nmen_MC58          EGYPTLDASALTLDTLLPPPDLPIFPESGVADISE  394
                       ********************:**************
```

2. Ng-LtgD (NEIS1212/NgO0626)

```
36675_NgO_P9-17      MEKRKILPLAICLAALSACTAMEARTPRANEAQAPRADEMKKESRPAFDA------AAVP    54
240_Nmen_MC58        MKKRKILPLAICLAALSACTAMEARPPRANEAQAPRAVEMKKESRPAFDAAAVFDAAAVP    60
4945_Nlac            MKKRKILPLAICLAALSACTAMETRPPRANEAQAPRADEMKKESRPTFD------AAAVP    54
                     *:*********************:* *********** ********:**       ****

36675_NgO_P9-17      VSDSGFAANANVRRFVDDEVGKGDFSQAEWQDFFDKAAYKADIVKIMHRPSTSRPWYVFR    114
240_Nmen_MC58        VSDSGFAANANVRRFVDDEVGKGDFSRAEWQDFFDKAAYKADIVKIMHRPSTSRPWYVFR    120
4945_Nlac            VSDSGFAANANVRRFVDDEVGKGDFSRAEWQDFFDKAAYKADIVKIMHRPSTSRPWYMFR    114
                     *************************:*******************************:**

36675_NgO_P9-17      TGNSGRAKFHGARRFYAENRAVIDDVAQKYGVPAELIVAIIGIETNYGKNTGSFRVADAL    174
240_Nmen_MC58        TGNSGKAKFRGARRFYAENRALIDDVAQKYGVPAELIVAVIGIETNYGKNTGSFRVADAL    180
4945_Nlac            TGNSGEAKFRGARRFYAENRALIDDVAQKYGVPAELIVAVIGIETNYGKNTGSFRVADAL    174
                     *****.***:***********:*****************:********************

36675_NgO_P9-17      ATLGFDYPRRAGFFQKELVELLKLAKEEGGDVFAFKGSYAGAMGMPQFMPSSYRKWAVDY    234
240_Nmen_MC58        ATLGFDYPRRAGFFQKELVELLKLAKEEGGDVFAFKGSYAGAMGMPQFMPSSYRKWAVDY    240
4945_Nlac            ATLGFDYPRRAGFFQNELVELLKLAKEEGGDVFAFKGSYAGAMGMPQFMPSSYRKWAVDY    234
                     ***************:********************************************

36675_NgO_P9-17      DGDGHRDIWGNVGDVAASVANYMKQHGWRTGGKMLVSATLAPGADVQAIIGEKTALTRTV    294
240_Nmen_MC58        DGDGHRDIWGNVGDVAASVANYMKQHGWRTGGKMLVSATLAPGADVQAIIGEKTALTRTV    300
4945_Nlac            DGDGHRDIWGNVGDVAASVANYMKQHGWRTGGKMLVSATLAPGADVQAIIGEKTALTRTV    294
                     ************************************************************

36675_NgO_P9-17      ADLKAYGIIPGETLADDEKAVLFKLETAPGVFEYYLGLNNFYTVWQYNHSRMYVTAVRDI    354
240_Nmen_MC58        ADLKAYGIIPGEELADDEKAVLFKLETAPGVFEYYLGLNNFYTVWQYNHSRMYVTAVRDI    360
4945_Nlac            ADLKAYGIIPGEELADDEKAVLFKLETAPSVFEYYLGLNNFYTVWQYNHSRMYVTAVRDI    354
                     ************ ****************.******************************

36675_NgO_P9-17      ANSLGGPGL*   363
240_Nmen_MC58        ANSLGGPGL*   369
4945_Nlac            ANSLGGPGL*   363
                     **********
```

The green highlighted amino acids belong to the binding sites of the enzymes that interact with the compounds.

**Ng-LdcA**																												
AtomNet (chain A amino acid residues)				ASP73	ARG76		ARG133	GLY134	GLY135		PHE164	SER165	ASP166	MET187	SER190	ASN236	SER238	VAL239	ASN261	VAL262	GLU264				ARG294			HIS331
Our active site analysis (chain A amino residues)																						TYR267	ARG268	ARG271		TYR302	ASP303	
(chain B amino acid residues)	GLY69	PHE70	GLU72			ARG103	ARG133		GLY135	TYR136		SER165				ASN236	SER238	VAL239	ASP261	VAL262	GLU264							
Molecular docking with substrate -binding amino acids	GLY69	PHE70	GLU72			ARG103	ARG133		GLY135	TYR136		SER165				ASN236	SER238	VAL239	ASP261	VAL262	GLU264	TYR267	ARG268	ARG271		TYR302	ASP303	
**Ng-LtgD**																												
AtomNet (chain A amino acid residues)	MET101	ILE157	GLU158	ASN160	ASN164	ARG184	TYR213	ALA214	GLN221	PHE222	MET223	SER226	TYR256	GLN340	TYR341	ASN342	HIS343	TYR347										
Our active site analysis (chain A amino residues)	MET101	ILE157	GLU158	ASN160	ASN164	ARG184	TYR213	ALA214	GLN221	PHE222	MET223	SER226	TYR256	GLN340	TYR341	ASN342	HIS343	TYR347										
Molecular docking with substrate -binding amino acids	MET101	ILE157	GLU158	ASN160	ASN164	ARG184	TYR213	ALA214	GLN221	PHE222	MET223	SER226	TYR256	GLN340	TYR341	ASN342	HIS343	TYR347										

Supplementary Figure 12. Alignment of active site amino acid residues for Ng-LdcA and Ng-LtgD, determined by AtomNet and our computational modelling. Yellow highlight denotes the shared amino acids identified by both algorithms.
