# Supplementary material for: Antimicrobial activity of compounds identified by artificial intelligence discovery engine targeting enzymes involved in Neisseria gonorrhoeae peptidoglycan metabolism
Source: Biol Res. 2024 Sep 5;57:62. doi: 10.1186/s40659-024-00543-9 (PMC11375863; doi:10.1186/s40659-024-00543-9)
Supplement: Supplementary file 2 — Additional file 2: Table S1. MIC and MBC values for all Ng-LdcA compounds tested against Neisseria gonorrhoeae P9-17. Table shows all compounds tested with their pre-screen values and titration values, MCULE ID, product URL and SMILES. The rows highlighted in yellow show the three compounds chosen for further computational modelling and in vitro studies. nd = no detectable killing; MIC, minimum inhibitory concentration; MBC, minimum bactericidal concentration; 41 is control DMSO; SMILES, simplified molecular-input line-entry system; ID and product url available from Mcule.com online drug discovery platform. S2. MIC and MBC values for all Ng-LtgD compounds tested against Neisseria gonorrhoeae P9-17. Table shows all compounds tested with their pre-screen values and titration values, MCULE ID, product URL and SMILES. The rows highlighted in yellow show the three compounds chosen for further computational modelling and in vitro studies. nd = no detectable killing; MIC, minimum inhibitory concentration; MBC, minimum bactericidal concentration; 66 is control DMSO; SMILES, simplified molecular-input line-entry system; ID and product url available from Mcule.com online drug discovery platform. S3. Specificity of compounds: summary of MIC and MBC for compounds tested against other bacteria. The top three Ng-LdcA and Ng-LtgD compounds tested against other bacteria. Values are generated from at least n = 2 MIC and MBC experiments. See Supplementary figures for titration curves for MIC and MBC experiments. [file 40659_2024_543_MOESM2_ESM.pdf]

Supplementary Table 1. MIC and MBC values for all Ng-LdcA compounds tested against *Neisseria gonorrhoeae* P9-17

Compound	MIC pre-screen (% kill at 50µM)	MIC$_{50}$ (µM)	MIC$_{>90}$ (µM)	MBC$_{50}$ (µM)	MBC$_{>90}$ (µM)	ID	Product URL	SMILES
1	18	nd	nd			MCULE-9351736864	https://mcule.com/P-9818202/	CC1N(CCC(OCC2OC(C3C(C4=CC=CC=C4)=NOC=3C)=NN=2)=O)C(=O)SC=1
2	5	nd	nd			MCULE-4772858662	https://mcule.com/P-458372661/	CCCCOC1=C(C(S(C2=CC=C(C)C=C2)(=O)=O)C#N)N=C2C(C=CC=C2)=N1
3	56	25-50	>50			MCULE-9332629201	https://mcule.com/P-12163838/	CC(C(C1ON=C(C2=CN=CC=C2)N=1)NS(C1=C(C)C(C)=C(C)C(C)=C1C)(=O)=O)C
4	92	12.5 - 25	25			MCULE-9919486569	https://mcule.com/P-12148322/	CN1C(=O)C2=C(N=CC(S(NCC(C3=CC=CC=C3)C3C4=C(C=CC=C4)NC=3)(=O)=O)=C2)N(C)C1=O
5	4	nd	nd			MCULE-6926757431	https://mcule.com/P-11175131/	CN(C(CCC1C(=O)NC(=O)NC=1C)=O)CC1=C(C)N(C2CS(=O)(=O)CC2)N=C1C
6	63	nd	>50			MCULE-3155097696	https://mcule.com/P-595019663/	S(=O)(=O)(N(CC)CC1ON=C(C(C)C)N=1)C1=CC=C(O1)C1ON=C(C)C=1C
7	85	12.5 - 25	25			MCULE-6138417371	https://mcule.com/P-11224951/	FC1=CC=C(CN(S(C2=CC3=C(NC(C(N3)=O)=O)C=C2)(=O)=O)C2CCCCC2)C=C1
8	0	nd	nd			MCULE-4760126721	https://mcule.com/P-6268600/	COC(C1C2=C(CCC2)SC=1NC(COC(CN1C2=C(C=CC=C2)N=C1C(F)(F)F)=O)=O)=O
9	0	nd	nd			MCULE-1967107616	https://mcule.com/P-11535196/	CN1C2=C(N(C(N(C2=O)CCCS(CC2N=C(C3OC=CC=3)SC=2)(=O)=O)=O)C)N=C1
10	0	nd	nd			MCULE-7249590981	https://mcule.com/P-21859926/	CC(CC1OC(C(S(C2NC(CC3=CC=CC=C3)=C(C)N=2)(=O)=O)C)=NN=1)C
11	13	nd	nd			MCULE-6504167942	https://mcule.com/P-10865396/	O=S(N1C(C2N3C(C=CC=C3)=NN=2)CCC1)(CCN1C2=C(C=CC=C2)N=C1)=O
12	0	nd	nd			MCULE-9857689135	https://mcule.com/P-600331448/	CCCCN1C(S(CC2ON=C(C3=COC=C3)N=2)(=O)=O)=NC(C)=C1C
13	3	nd	nd			MCULE-6790386579	https://mcule.com/P-594938541/	S(=O)(=O)(NC(CC(C)(C)C)C1=CC=CN=C1)C1=CC=C(O1)C1=NN=C(C2CC2)O1
14	6	nd	nd			MCULE-6988194119	https://mcule.com/P-594892614/	S(=O)(=O)(NC(CC(C)C)C1N(C)N=CN=1)C1C(C)=NOC=1C
15	94	25 - 50	25 - 50			MCULE-1832366245	https://mcule.com/P-10974822/	CC(C(C=C1C(C)(C)C2=C(C=CC=C2)N1C)=O)OC(CN1C2=C(N(C(N(C2=O)C)=O)C)N=C1)=O
16	93	<1.56	3.125 - 6.25	0.195 - 0.39	0.39	MCULE-3643079078	https://mcule.com/P-8020596/	ClC1=CC=C(C=C2C(=O)NC(=CC(C3OC=CC=3)=O)S2)C=C1

17	0	nd	nd			MCULE-9148071268	https://mcule.com/P-10971873/	CC1C(CCC(OCC2N(C)C(=O)N(C)C(=O)C=2)=O)=C(C)N2C(=NC(C(F)(F)F)=N2)N=1
18	0	nd	nd			DMSO	0	0
19	91	25 - 50	>50			MCULE-8209982886	https://mcule.com/P-11946020/	CC(S(CC1ON=C(C2=CC(C)=CC=C2)N=1)(=O)=O)C1ON=C(C2CC2)N=1
20	0	nd	nd			MCULE-2962671571	https://mcule.com/P-600343598/	CCC1N(C)C(CS(CC2N=C(C3=C(F)C=CC=C3F)OC=2)(=O)=O)=NN=1
21	0	nd	nd			MCULE-1702727320	https://mcule.com/P-5321235/	COC1=C(OC)C=C(N(=O)=O)C(C(OC(C(NC2SC=C(C3=CC(F)=C(F)C=C3)N=2)=O)C)=O)=C1
22	15	nd	nd			MCULE-7881428223	https://mcule.com/P-10975931/	CN1C(=O)N(C)C(=O)C=C1CN1C(=O)N2C(CCC2)=C(C#N)C1=O
23	54	12.5 -25	>50			MCULE-1205028784	https://mcule.com/P-5239481/	CC(C1C(C)=C(C(OCC(C2=CC3=C(CC4=C3C=CC=C4)C=C2)=O)=O)NC=1C)=O
24	19	nd	nd			MCULE-2434830919	https://mcule.com/P-595010949/	C1(=CN(C)C(N(C)C1=O)=O)S(=O)(=O)N1CCC(C1)C1C=CC=C(OC)C=1OC
25	36	nd	nd			MCULE-7677128589	https://mcule.com/P-594946868/	S(=O)(=O)(N1CCC[C@H]1C1ON=C(C(C)C)N=1)C1=CN(C)C(N(C)C1=O)=O
26	18	nd	nd			MCULE-3534323006	https://mcule.com/P-600403992/	CCCN1C2=C(N(C(N(C2=O)CC(C(F)(F)F)O)=O)C)N=C1
27	11	nd	nd			MCULE-1634880159	https://mcule.com/P-459326978/	CC(C1OC(COC2C(S(N3CC(CO)C3)(=O)=O)=CC=CC=2)=NN=1)C
28	8	nd	nd			MCULE-6354205207	https://mcule.com/P-32872635/	CCC1ON=C(C2N(C(CN3C(=O)N(C)C(=O)C=C3)=O)CCC2)N=1
29	97	12.5 - 25	>50			MCULE-3442536716	https://mcule.com/P-457696925/	CC1=C(C(C(C(C2=CC=C(C3N=C(C(F)(F)F)ON=3)C=C2)=O)C#N)=O)OC=C1
30	96	25 -50	25 -50			MCULE-5005245930	https://mcule.com/P-457740500/	O=C(C1=C2C(NC(O2)=O)=CC=C1)OCC#CC1=CC=CC=C1
31	1	nd	nd			MCULE-7446280834	https://mcule.com/P-600211486/	CN1C(=O)N(C)C(=O)C(CC2C(=O)N(C)C(=O)N(C)C=2)=C1
32	0	nd	nd			MCULE-3700728379	https://mcule.com/P-459296382/	CC1=C(S(NC2=CC(N=S(C)(C)=O)=CC=C2)(=O)=O)C(N)=NO1
33	0	nd	nd			MCULE-9249795968	https://mcule.com/P-11156532/	CN1C(=O)N(C)C(=O)C(C#N)=C1OC1=CC=C(S(C)(=O)=O)C=C1
34	0	nd	nd			MCULE-4762903767	https://mcule.com/P-10150002/	O=C(OCC1N=C2N(C3=C(S2)C=CC=C3)C(=O)C=1)CN1C(=O)N(CC2=CC=CC=C2)C=CC1=O
35	97	0	0			MCULE-2482124158	https://mcule.com/P-10147603/	ClC1=CC2=C(N(C(O2)=O)CC2OC(C3=CC=CC=C3)=CN=2)C=C1

36	88	12.5 - 25	>50			MCULE-2394732480	https://mcule.com/P-10339105/	CN(S(C1=C(C)N(CC2ON=C(C3=CC(Cl)=CC=C3)N=2)N=C1C)(=O)=O)CC1OC=CC=1
37	91	1.56 - 3.125	6.25	1.56 - 3.125	6.25-12.5	MCULE-8421686119	https://mcule.com/P-9818716/	FC1=CC2=C(OC(C3=CSC=C3)OC2)C(COC(CN2C(=O)C3=C(C4=C(O3)C=CC=C4)N=C2)=O)=C1
38	10	nd	nd			MCULE-7756290556	https://mcule.com/P-457788470/	CC1=CN(CCC2ON=C(C(S(C3=CC=C(Br)C=C3)(=O)=O)(C)C)N=2)N=C1
39	93	25 - 50	>50			MCULE-9863170854	https://mcule.com/P-5517514/	O=C(NC1=C(OC2=CC=CC=C2)C=CC=C1)COC(CC1C2=C(C=CC=C2)C(=O)NN=1)=O
40	0	nd	nd			MCULE-1257979770	https://mcule.com/P-32956551/	CCC(S(C)(=O)=O)(CN1C(=O)C2=C(C=CC=C2)NC1=O)CC
41	1	nd	nd			DMSO	0	0
42	60	25 - 50	>50			MCULE-4794825564	https://mcule.com/P-33073764/	CCC1=CC=C(S(N2C(C3N=C(C4=CN(C)N=C4)ON=3)COCC2)(=O)=O)C=C1
43	19	nd	nd			MCULE-7241883322	https://mcule.com/P-459104401/	CC(N1N=CC(S(N2C(C3=NOC=N3)CCCCC2)(=O)=O)=C1)C
44	0	nd	nd			MCULE-7741400783	https://mcule.com/P-33016419/	COCCC1N=C(CN2C(=O)N=CN2C2=C(C)C=CC(C)=C2)ON=1
45	0	nd	nd			MCULE-3991542810	https://mcule.com/P-11754110/	CCN(C(CS(CC1N(C)C(=O)N(C)C(=O)C=1)(=O)=O)=O)C1CC1
46	97	25 - 50	25 - 50			MCULE-9852656674	https://mcule.com/P-595008919/	S(=O)(=O)(N1CCCC(C1)C1C=CNN=1)C1=C(C)OC(C2=NOC(C(F)(F)F)=C2)=C1
47	0	nd	nd			MCULE-2716262305	https://mcule.com/P-448873668/	C(CN1C=NC2N(C)C(=O)N(C)C(=O)C=21)(=O)N1CCC(CC1)N1N=C(C(=O)OCC)C=C1C(C)(C)C
48	96	12.5 - 25	25 - 50			MCULE-2660099127	https://mcule.com/P-10094808/	CCCCN1C(=O)C=CC(C(OCC2ON=C(C3=CC(Br)=CC=C3)N=2)=O)=N1
49	4	nd	nd			MCULE-2449068842	https://mcule.com/P-10864901/	CN1C(=O)C2=C(N=CN2CCS(NC2C3=C(C=CC=C3)OCC2)(=O)=O)N(C)C1=O
50	93	>50	>50			MCULE-1278281161	https://mcule.com/P-11356776/	COC(C(C(C(F)(F)F)(C1N(C)C=CN=1)O)(COC(C1=CC(Cl)=CC=C1)=O)O)=O
51	49	nd	nd			MCULE-7461218401	https://mcule.com/P-11170337/	COC(C1=CN(CC(C2=C(C)N(C3SC=CN=3)C(C)=C2)=O)C(=O)C=C1)=O
52	0	nd	nd			MCULE-4528910265	https://mcule.com/P-9942036/	ClC1=CC(Cl)=C(C(COC(C2C3=C(C=CC=C3)C(=O)NN=2)=O)=O)C=C1
53	79	25 - 50	>50			MCULE-7781936176	https://mcule.com/P-5342233/	FC1=C(F)C=C(C(COC(CN2C(=O)C3=C(C=CC=C3)C(=O)N2)=O)=O)C=C1
54	12	nd	nd			MCULE-2558307031	https://mcule.com/P-595013307/	S(=O)(=O)(N(C)CC1=NN=CN1CCOC)C1C(C)=NOC=1C

55	14	nd	nd			MCULE-8816785241	https://mcule.com/P-600378448/	CC1=C(CS(C2N3C(CCCCC3)=NN=2)(=O)=O)N=C(C2OC=CC=2)O1
56	65	12.5 - 25	>50			MCULE-3414448315	https://mcule.com/P-10729634/	COC1=CC(C2C(C(OCC3ON=C(C)C=3)=O)=CN(C3=CC=CC=C3)N=2)=CC=C1
57	14	nd	nd			MCULE-1908373429	https://mcule.com/P-6266539/	O=C(C1=NN(C2=CC=CC=C2)C(C2=CC=CC=C2)=N1)OCC1OC(C2=CC=CC=C2)=CN=1
58	15	nd	nd			MCULE-2474288924	https://mcule.com/P-5843518/	COC1=CC(CNC(COC(CNC(C2=CC=C(C3=CC=CC=C3)C=C2)=O)=O)=O)=CC=C1
59	85	25 - 50	>50			MCULE-7042090559	https://mcule.com/P-11219637/	CS(C1=CC(C(OCC2ON=C(COC3=CC=C(F)C=C3)N=2)=O)=C(Cl)C=C1)(=O)=O
60	52	25 - 50	>50			MCULE-9681378024	https://mcule.com/P-32618626/	CC(CC1ON=C(CS(C(C2ON=C(C(C)C)N=2)C)(=O)=O)N=1)C
61	3	nd	nd			MCULE-2816392274	https://mcule.com/P-6389920/	CCCCN1C2=C(N(C=N2)CC2OC=CC=2)C(=O)N(CC2N=C3N(C=CC(=C3)C)C(=O)C=2)C1=O
62	79	50	>50			MCULE-6395370829	https://mcule.com/P-10084391/	CN(C(CN1C(=O)C2=C(N=CN2C2CCCC2)N(CC2=CC=CC=C2)C1=O)=O)CC1OC(C)=CC=1
63	53	6.25 - 12.5	25			MCULE-7018519984	https://mcule.com/P-9632516/	CC1N(C2CS(=O)(=O)CC2)C(C)=C(C(CN2C(=O)C=CC(S(N3CCCC3)(=O)=O)=C2)=O)C=1
64	83	12.5 - 25	>50			MCULE-7246259705	https://mcule.com/P-11196998/	OC1=C(C(C2=CN(C3=CN=CC=C3)C(=O)C(C(C3=CC=CC=C3)=O)=C2)=O)C=CC=C1
65	36	nd	nd			MCULE-1178547415	https://mcule.com/P-458984962/	CC1=C(Br)C(C(NCC(C2OC=CC=2)=O)=O)=CC=C1
66	39	nd	nd			MCULE-9206759588	https://mcule.com/P-33099469/	CCC1C(C(C)=O)=C(C)NC=1C(CN1C2C(OC(N=2)=O)=CC=C1)=O
67	43	nd	nd			MCULE-9695341938	https://mcule.com/P-457959971/	CC1=C(C(C(C(C2=COC=C2)=O)C#N)=O)C2=C(C=CC=C2)N1
68	18	nd	nd			MCULE-1425751501	https://mcule.com/P-594958961/	S(=O)(=O)(N(C1CC1)CC1C(C)=NN(C)C=1C)C1=CC=C(C2=CC=NO2)O1
69	59	<1.56	3.125-6.25	0.78-1.56	1.56	MCULE-7440025223	https://mcule.com/P-32383805/	CC1C(=O)C2=C(C(C(OCC(NC34CC5CC(C3)CC(C5)C4)=O)=O)=CC=C2)OC=1C1=CC=CC=C1
70	50	6.25	>50			MCULE-1941420343	https://mcule.com/P-9714277/	COC1C(C(OCC(C2=CC3=C(CCC3)C=C2)=O)=O)=NN(C2=CC=CC=C2)C=1
71	20	nd	nd			MCULE-9771713959	https://mcule.com/P-9805992/	O=C(C1C2=C(C=CC=C2)NN=1)OCC1NC(=O)C2=C(SC=C2C2OC=CC=2)N=1
72	33	nd	nd			MCULE-1973838782	https://mcule.com/P-594887360/	C12=NON=C1C(C)=CC(C)=C2S(NCCCN1C=CN=C1C(C)C)(=O)=O
73	41	nd	nd			MCULE-9885319855	https://mcule.com/P-10116693/	CCOC(C1=C(CN2C(=O)C3=C(SC=C3)N=C2)OC=C1)=O

74	20	nd	nd			MCULE-5826070097	https://mcule.com/P-5640739/	ClC1=CC(NC(COC(C2OC3=C(C=CC=C3)C(=O)C=2)=O)=O)=C(C#N)C=C1

Table shows all compounds tested with their pre-screen values and titration values, MCULE ID, product URL and SMILES. The rows highlighted in yellow show the three compounds chosen for further computational modelling and in vitro studies.

nd = no detectable killing; MIC, minimum inhibitory concentration; MBC, minimum bactericidal concentration; 41 is control DMSO; SMILES, simplified molecular-input line-entry system; ID and product url available from Mcule.com online drug discovery platform.

Supplementary Table 2. MIC and MBC values for all Ng-LtgD compounds tested against *Neisseria gonorrhoeae* P9-17

Compound no	MIC pre-screen (% kill at 50µM)	MIC_{50} (µM)	$MIC_{>90}$ (µM)	MBC_{50} (µM)	$MBC_{>90}$ (µM)	ID	Product URL	SMILES
1	0	nd	nd			MCULE-5402205416	https://mcule.com/P-33028381/	CCCC1N=C(CNC(C2=CC3=C(NC(CC3)=O)C=C2)C)ON=1
2	0	nd	nd			MCULE-1826930811	https://mcule.com/P-10242357/	CCN(CC1OC2=C(C=CC=C2)N=1)CC(NC1CS(=O)(=O)CC1)=O
3	0	nd	nd			MCULE-8525112445	https://mcule.com/P-21832333/	CN1CC(CNCC2C(C3=CN=CC=C3)=NN(C)C=2)OCC1
4	74	6.25 -12.5	12.5 - 25			MCULE-9875835553	https://mcule.com/P-457847456/	CC1=C(Br)C=CC(NC(COC(C2C(=O)NC=CC=2)=O)=O)=C1
5	0	nd	nd			MCULE-7566373228	https://mcule.com/P-600311154/	CN1C(=O)NC(=O)C(OC2=C(Cl)C=C(F)C=C2)=C1N
6	0	nd	nd			MCULE-4238612868	https://mcule.com/P-32643591/	OC(CN1N=CC=C1)CN1CCN(CC2N=C3N(C=CC=C3)C=2)CC1
7	0	nd	nd			MCULE-4095105661	https://mcule.com/P-458887553/	Cl.CC(C(NCC1C=NC(N2C(C)=CC(C)=N2)=CC=1)=O)N
8	0	nd	nd			MCULE-3649936070	https://mcule.com/P-32874605/	CC1C(N(C)C)CN(C(C2OC(CN3C(C)=CC(C)=N3)=CC=2)=O)C1
9	0	nd	nd			MCULE-6219229542	https://mcule.com/P-457992516/	NC(CN(C1CCCC1)CC1ON=C(C2OC=CC=2)N=1)=O
10	0	nd	nd			MCULE-4914010762	https://mcule.com/P-21979864/	CC1=C(CC2OC(CN3CCN(CC4=NC=CC=C4)CC3)=NN=2)C=CC=C1
11	8	nd	nd			MCULE-2561383416	https://mcule.com/P-12017997/	CN1CCN(CC2=CC(NC(CCCC3ON=C(C)N=3)=O)=CC=C2)CC1
12	8	nd	nd			MCULE-2806656309	https://mcule.com/P-32528471/	CN(CC1N(C)C(=O)N(C)C(=O)C=1)CC(NC(C)(C)C)=O
13	1	nd	nd			MCULE-1981814214	https://mcule.com/P-32801281/	CC1SC=C(CN2CCN(C(C3OC(Cl)=CC=3)=O)CCC2)N=1
14	100	6.25 -12.5	>50			MCULE-7832625130	https://mcule.com/P-7720070/	C1(CN/C(=N\C2=CC=C(C=C2)C(C)C)/NC(=O)C2C=CC=CC=2C)=CN(N=C1C)CCC
15	0	nd	nd			MCULE-7517252584	https://mcule.com/P-32767020/	CCN1CCN(C(C2=C(C)N=C(C3OC=CC=3)C=C2)=O)CC1
16	90	12.5 -25	>50			MCULE-5999099089	https://mcule.com/P-8349806/	CCOC(C1=C(COC2=C(C(N)=O)C=CC=C2)C2=C(C=CC=C2)O1)=O

17	0	nd	nd			MCULE-2573698990	https://mcule.com/P-11027636/	O=C(N1CCC2(OC3=C(C=CC=C3)C(=O)C2)CC1)CN1C2C(N(C3=CC=CC=C3)C(=O)C2N=N1)=O
18	0	nd	nd			MCULE-6528144389	https://mcule.com/P-32878762/	CC(N1C(=O)C=C2C(CCN(C2)CC2ON=C(C3OC=CC=3)N=2)=N1)C
19	0	nd	nd			MCULE-8580878696	https://mcule.com/P-600339681/	CC(N(C(C(NCCN1N=CC=C1)C)=O)C(C)C)C
20	0	nd	nd			MCULE-6951466004	https://mcule.com/P-32878607/	CC(C(NC1ON=C(C)C=1)=O)N1CC2C(=NN(C(C=2)=O)C)CC1
21	0	nd	nd			MCULE-1697322737	https://mcule.com/P-21570952/	CC(N1CCOCC1)CNC(CCCN1C2=C(C=CC=C2)OC1=O)=O
22	0	nd	nd			S-614260667	https://mcule.com/S-614260667/	Cl.COC1=C(C)C(C)=C(C2OC(C)=C(CN3C(C)CNCC3)N=2)C=C1
23	4	nd	nd			MCULE-7246389006	https://mcule.com/P-32940873/	NCC1CCN(C(C2=C(C3=CN=CN=C3)C=CC=C2)=O)CC1.Cl.Cl
24	7	nd	nd			MCULE-2904736358	https://mcule.com/P-32547417/	CN(C(C1OC=CC=1)CNCC1=C(C)OC(C2=CC=CC=C2)=N1)C
25	0	nd	nd			MCULE-6725261859	https://mcule.com/P-32664274/	CC1C(CNCC(C2OC=CC=2)N2CCCCC2)=CNN=1
26	0	nd	nd			S-614260663	https://mcule.com/S-614260663/	Cl.CC1=CC=C(C2=NOC(CN(C(C3N=C(CN)OC=3)=O)CC)=N2)C=C1
27	0	nd	nd			MCULE-6431097705	https://mcule.com/P-32518153/	CC1=NOC(CN2CCN(C(C3=C(C)ON=C3C)=O)CC2)=C1
28	0	nd	nd			MCULE-7609242771	https://mcule.com/P-600429628/	CC1NCCN(C(C2=CC3=C(N(C(C)=O)CC3)C=C2)=O)C1
29	0	nd	nd			MCULE-7954709626	https://mcule.com/P-457753637/	CN(CC1ON=C(C(NC(C2=CC(N(=O)=O)=C(C)C=C2)=O)(C)C)N=1)C
30	0	nd	nd			MCULE-6335324481	https://mcule.com/P-9396903/	CCN(C(C1=CC=CC=C1)CNC(C1=C(O)N=CC=C1)=O)CC
31	0	nd	nd			MCULE-6254257134	https://mcule.com/P-21847301/	CC1N(C(C2=CN3C(=NN=C3C)C=C2)=O)CCN(C)C1
32	0	nd	nd			MCULE-6620563568	https://mcule.com/P-10623881/	CC(C1=CC(OC2=C(N(=O)=O)N3C(SC=C3)=N2)=CC=C1)=O
33	0	nd	nd			MCULE-3684645404	https://mcule.com/P-600283517/	Cl.CN1CCN(C(CNCC2C(C(C)(C)C)=NNC=2)(C)C)CC1
34	0	nd	nd	DMSO			0	0
35	10	nd	nd			MCULE-6641484659	https://mcule.com/P-32985849/	CCOC(C1=C(NC(C2=CC(Cl)=CC=C2)=O)OC(CC)=N1)=O

36	7	nd	nd			MCULE-9988336810	https://mcule.com/P-32996754/	CCN1C(C)CN(C(C2C3=C(C=CC=C3)NN=2)=O)C(C)C1
37	52	25 - 50	>50			MCULE-3971539248	https://mcule.com/P-21922470/	CCSC1CN(C(C2ON=C(C3NC=NN=3)N=2)C)CCCC1
38	8	nd	nd			MCULE-1215320224	https://mcule.com/P-600337653/	C(C1OC(C2OC=CC=2)=NN=1)NC1CN2C(=NC=N2)CC1
39	7	nd	nd			S-614260665	https://mcule.com/S-614260665/	Cl.Cl.NC1CCN(CC2=C(CN3N=CC=C3)C=CC=C2)C1
40	7	nd	nd			MCULE-3459085795	https://mcule.com/P-458427368/	CCN1C2=C(C(=CC=C2)F)N=C1CN(CC(N(C)C)=O)C
41	18	nd	nd			MCULE-2352041767	https://mcule.com/P-32754034/	CC(C1=CC=CC=C1)N(C(CN1CC2N(C=CN=2)CC1)=O)C
42	5	nd	nd			S-614260664	https://mcule.com/S-614260664/	Cl.CC1(CC(NC(CN)=O)C2C=NN(C3=CC=CC=C3)C=2C1)C
43	30	nd	nd			MCULE-1366616997	https://mcule.com/P-600390871/	CCN1N=CC(CN2CCN(CC3=NC=C(Cl)C=C3)CC2)=C1
44	46	nd	nd			MCULE-1868392968	https://mcule.com/P-457978765/	CN(CC1=C(F)C=CC(C#N)=C1)CC1=NOC(C2OC=CC=2)=C1
45	83	1.56 - 3.125	6.25 - 12.5	3.125	6.25	MCULE-8306618236	https://mcule.com/P-5967046/	COC1=CC=C(C(C(OC(C2=C(C(C3=CC=CC=C3)=O)C=CC=C2)=O)C)=O)C=C1
46	58	25 - 50	>50			MCULE-5682808554	https://mcule.com/P-33034603/	CC1=C(CNCC2=CN(C3=CC=C(Cl)C=C3)N=C2)N=CO1
47	9	nd	nd			MCULE-5261090709	https://mcule.com/P-600436135/	CC(C1CN(C(C2C(C)=NN(C3C=CC=C3)C=2)=O)CCC1)N
48	15	nd	nd			MCULE-1769855945	https://mcule.com/P-32671771/	FC1=C(COC(C2C(=O)NC3=C(C(CCC3)=O)C=2)=O)C=CC=C1
49	6	nd	nd			MCULE-7521206811	https://mcule.com/P-600438842/	CCN(C(C1OCCNC1)=O)CC1NC(=O)C2C=CC=CC=2N=1.Cl
50	10	nd	nd			MCULE-7518684248	https://mcule.com/P-600435539/	NC1CN(C(C2C=CC=C(N3N=C(C(F)(F)F)C=C3)C=2)=O)CC1.Cl
51	9	nd	nd			MCULE-3628288911	https://mcule.com/P-594735136/	S(=O)(=O)(N1CCCCC1)N1CCCN(CC1)C(=O)C1=CC=CC(=O)N1
52	96	3.125 - 6.25	12.5 - 25	3.125	25	MCULE-9819938275	https://mcule.com/P-5947842/	CC1=C(C2N=C(COC(C3=C(N(=O)=O)C=CC=C3)=O)ON=2)C=CC=C1
53	4	nd	nd			MCULE-8307907525	https://mcule.com/P-600437473/	CC1NC(C)CN(C(CN2C(=O)N(C)C(=O)C=C2)=O)C1.Cl
54	4	nd	nd			MCULE-8375210046	https://mcule.com/P-12216557/	CC(CN1CCN(C(CN2C(=O)C3=C(C=CC=C3)N=C2C)=O)CC1)O

55	0	nd	nd			MCULE-2226821570	https://mcule.com/P-32808078/	CN1C(CN2CCN(CC3=CN(C4=CC=CC=C4)N=C3)CC2)=NC=C1
56	2	nd	nd			MCULE-5195787059	https://mcule.com/P-594719305/	C12(C3=CC=CC=C3)CN3CCN(CC(C3)C1NC(=O)C1=C(C)OC=N1)C2
57	4	nd	nd			MCULE-6181275035	https://mcule.com/P-600330792/	CC(C(N1N=CC(C)=C1)C)NCC1OC2=C(C=CC=C2)N=1
58	0	nd	nd			MCULE-9227482718	https://mcule.com/P-21795540/	CN(C(CN1C(C2N=C(C)ON=2)CCC1)=O)CC1OC(C)=CC=1
59	55	25	>50			MCULE-6452775739	https://mcule.com/P-32788693/	CC(C1=C(F)C=C(F)C=C1)N1CCN(CC2OC(C)=C(C)N=2)CC1
60	19	nd	nd			MCULE-1410819308	https://mcule.com/P-600312824/	CCN1C(CN2C(C)CN(CC3=CN=CC=C3)CC2)=NC=C1
61	11	nd	nd			MCULE-9733577488	https://mcule.com/P-10013851/	COC(C1=C(C=C(C2SC3=C(C=CC=C3)N=2)CC(O)=O)C=CC=C1)=O
62	11	nd	nd			MCULE-5377608973	https://mcule.com/P-458826903/	CN(C1CC1)CCN(C(C1=C2C(OC(N2)=O)=CC=C1)=O)C
63	6	nd	nd			DMSO	0	0
64	14	nd	nd			MCULE-9989877758	https://mcule.com/P-458979492/	COCC1N=C(C(N2CCC(NC(C3=C(C)C=CO3)=O)CC2)C)ON=1
65	11	nd	nd			MCULE-3210475007	https://mcule.com/P-10728011/	CC(COC(C1C2=C(C=CC=C2)C(=O)NN=1)=O)C
66*	3	nd	nd			MCULE-5834215945	https://mcule.com/P-458647344/	CN(C(C1=CN(C)N=C1)CNC(C1=C2C(CCC2)=CO1)=O)C
67	3	nd	nd			S-614260662	https://mcule.com/S-614260662/	Cl.Cl.N[C@H](C(N1CCC(C2N(CC3=CC=NC=C3)C=CN=2)CC1)=O)CC1N=CNC=1
68	81	0	0			MCULE-3283592052	https://mcule.com/P-6016194/	CC(C1C(C)=C(C(COC(C2=C(Cl)C=CC=C2F)=O)=O)NC=1C)=O
69	83	3.125 - 6.25	6.25 - 12.5	3.125	12.5	MCULE-3265516242	https://mcule.com/P-21165293/	ClC1C=C(C(OC(C2=CC=CC=C2)C(C2=CC=CC=C2)=O)=O)NC=1
70	7	nd	nd			MCULE-7097731212	https://mcule.com/P-32809847/	CCN1C(C2N(C(C3=CC4=C(NC=N4)C=C3)=O)CCC2)CCC1
71	38	nd	nd			MCULE-9329689605	https://mcule.com/P-32643891/	CCN(CC1N=C(C2OC(C)=CC=2)SC=1)CC(N(C)C)=O
72	48	nd	nd			MCULE-9756412694	https://mcule.com/P-32814380/	CN1C2=C(CN(CC2)CC2OC3=C(C=CC=C3)N=2)N=C1
73	30	nd	nd			MCULE-5347049686	https://mcule.com/P-600377357/	CC(NC(C1=CC2=C(N(C=N2)C)N=C1)=O)CN1CCN(C)CC1

74	56	0	0			MCULE-1674205400	https://mcule.com/P-32883338/	CCCN1CCN(C2CCN(CC3OC(C)=CN=3)CC2)CC1
75	12	nd	nd			MCULE-1384664172	https://mcule.com/P-33100381/	CC1CCN(C(CN2C(C3ON=C(C)N=3)COCC2)=O)CC1
76	99	12.5 - 25	>50			MCULE-3008309249	https://mcule.com/P-32643333/	O=N(C1=C(OC2OC3=C(C=CC=C3)N=2)C=CC(Cl)=C1)=O
77	28	nd	nd			MCULE-2099181054	https://mcule.com/P-21985372/	CC1=C(Br)C(O)=C(C(OCC(N2CCCC2)=O)=O)C=N1
78	97	6.25 - 12.5	12.5 - 25			MCULE-3200368508	https://mcule.com/P-600434801/	CC1N(C2C=CC(Br)=CC=2)N=C(C(N2CC(CN)CC2)=O)C=1
79	27	nd	nd			MCULE-5574590708	https://mcule.com/P-458896197/	Cl.NCC1=NC(C2CC2)=C(C(N2C3=C(C=CC=C3)CC2)=O)C=C1
80	23	nd	nd			MCULE-1870030172	https://mcule.com/P-600441255/	CC([C@@H](C(NC1=CN(CC2=CC=NC=C2)N=C1)=O)N)(C)C.Cl.Cl
81	23	nd	nd			MCULE-8974149791	https://mcule.com/P-600438137/	CNCC1N(C(C2=CN3C(=NC=C3)C=C2)=O)CCC1
82	14	nd	nd			MCULE-4178783632	https://mcule.com/P-11570121/	CN(C(C1=CSC=C1)CNCC1=CC(N(=O)=O)=CC=C1)C
83	12	nd	nd			MCULE-5833742587	https://mcule.com/P-600239408/	CC(N1N=CC(CNCC2=C(N3N=CC=C3)N=CC=C2)=C1)(C)C.Cl
84	24	nd	nd			MCULE-3789015380	https://mcule.com/P-32547440/	CN1CCN(C(CC2=C3C(C=CC=N3)=CC=C2)=O)CC1

Table shows all compounds tested with their pre-screen values and titration values, MCULE ID, product URL and SMILES. The rows highlighted in yellow show the three compounds chosen for further computational modelling and *in vitro* studies.

nd = no detectable killing; MIC, minimum inhibitory concentration; MBC, minimum bactericidal concentration; 66 is control DMSO; SMILES, simplified molecular-input line-entry system; ID and product url available from Mcule.com online drug discovery platform.

Supplementary Table 3. Specificity of compounds: summary of MIC and MBC for compounds tested against other bacteria

Organism	Target	Compound	MIC_{50} (µM)	MIC_{90} (µM)	MBC_{50} (µM)	MBC_{90} (µM)
Lactobacillus gasseri	LcdA	16	>50	>50	>50	>50
		37	>50	>50	>50	>50
		69	>50	>50	50	>50
	LtgD	45	>50	>50	12.5	>50
		52	>50	>50	>50	>50
		69	>50	>50	12.5	12.5
Pseudomonas aeruginosa	LcdA	16	>50	>50	>50	>50
		37	>50	>50	>50	>50
		69	>50	>50	>50	>50
	LtgD	45	>50	>50	>50	>50
		52	>50	>50	>50	>50
		69	>50	>50	>50	50
Staphylococcus aureus NCTC8325.4	LcdA	16	>50	>50	>50	>50
		37	>50	>50	>50	>50
		69	>50	>50	>50	>50
	LtgD	45	>50	>50	>50	>50
		52	>50	>50	>50	>50
		69	50	>50	>50	>50
S. aureus 6517	LtgD	45	>50	>50	>50	>50
		52	>50	>50	>50	>50
		69	>50	>50	>50	>50
S. aureus 29213	LtgD	45	>50	>50	>50	>50
		52	>50	>50	>50	>50
		69	>50	>50	>50	>50
S. capitis 09E395	LtgD	45	>50	>50	>50	>50
		52	>50	>50	>50	>50
		69	>50	>50	>50	>50
S. epidermidis 12228	LtgD	45	>50	>50	>50	>50
		52	>50	>50	>50	>50
		69	>50	>50	>50	>50

The top three Ng-LdcA and Ng-LtgD compounds tested against other bacteria. Values are generated from at least n=2 MIC and MBC experiments. See Supplementary figures for titration curves for MIC and MBC experiments.
